# The representation of emotion knowledge across development

**DOI:** 10.1111/cdev.13716

**Published:** 2021-11-25

**Authors:** Kristina Woodard, Martin Zettersten, Seth D. Pollak

**Affiliations:** ^1^ Department of Psychology University of Wisconsin–Madison Madison Wisconsin USA; ^2^ Department of Psychology Princeton University Princeton New Jersey USA

## Abstract

The present study examined how children spontaneously represent facial cues associated with emotion. 106 three‐ to six‐year‐old children (48 male, 58 female; 9.4% Asian, 84.0% White, 6.6% more than one race) and 40 adults (10 male, 30 female; 10% Hispanic, 30% Asian, 2.5% Black, 57.5% White) were recruited from a Midwestern city (2019–2020), and sorted emotion cues in a spatial arrangement method that assesses emotion knowledge without reliance on emotion vocabulary. Using supervised and unsupervised analyses, the study found evidence for continuities and gradual changes in children's emotion knowledge compared to adults. Emotion knowledge develops through an incremental learning process in which children change their representations using combinations of factors—particularly valence—that are weighted differently across development.

**Abbreviations**
ESG: Evaluative Space Grid
MDS: multidimensional scaling
OSF: Open Science Framework
SpAM: spatial arrangement method

Emotions are a rich source of information that children learn to use when formulating predictions about what is likely to occur in their environments. For example, facial movements from others, in combination with other contextual information, help children understand whether their actions are approved of by their social partners or caregivers, whether they should approach or avoid persons, and whether an environment is safe. Children's acquisition of the ability to make use of emotion cues develops so rapidly that the considerable learning involved can appear seamless, masking potentially important changes across development (Pollak et al., 2019; Ruba & Pollak, 2020). The present study addresses how children think about and organize the perceptual input of facial configurations associated with emotions.

The longest standing theory about the structure of emotion from early infancy was proposed by Bridges (1932), who observed that children begin by fluctuating between a resting state of calm with punctuated states of distress. This view was the basis of contemporary theories that human understanding of emotions begins with differentiation between distress/lack of distress, and becomes elaborated over time into fine‐grained emotion categories (Nook & Somerville, 2019; Widen, 2013; Widen & Russell, 2008, 2010a). These theories leave unresolved how children organize and represent the range of perceptual features they encounter and how this becomes elaborated over development.

The concepts most frequently used to refer to the initial building blocks of emotion experience and perception are valence and arousal (e.g., Bliss‐Moreau et al., 2020; Russell, 2003). Valence (positivity/negativity) can be conceptualized either as bipolar (a single scale from positive to negative with a neutral midpoint) or bivariate (two orthogonal scales of positivity and negativity; Larsen et al., 2009; Mattek et al., 2020). The dimension of arousal captures low to high activity or engagement. Other theories propose that key physical features such as open or closed mouths form not only the basis of face perception, but also emotion reasoning (Caron et al., 1985). And still, other views maintain that children have a rudimentary sense of a limited set of emotion categories that they use to understand facial configurations (Izard, 2007; Leppänen & Nelson, 2009). Historical and anthropological perspectives have emphasized language as key building block of emotion (Harré, 1986; Lutz & White, 1986), a view that has recently re‐emerged (Hoemann et al., 2019; Lindquist, 2021; Nook et al., 2020).

The primary challenge to understanding how children think about various emotion cues concerns the difficulty in accurately assessing what children are perceiving when they are exposed to stimuli such as facial configurations (Barrett et al., 2019). Much of the data used to understand the structure of young children's emotion knowledge relies upon children's production and comprehension of emotion labels (see Ruba & Pollak, 2020, in particular the sections on verbal‐response paradigms). The most commonly used approaches in this field involve asking children to generate a verbal label to describe a facial stimulus such as “*What is this person feeling?*” (Nelson & Russell, 2011; Widen & Russell, 2003). Other common methods include sorting images into labeled piles (Hoemann et al., 2021; Matthews et al., 2020), confirming whether labels match an image displayed with prompts such as “*Is this person feeling sad?*” (Widen & Russell, 2008), or selecting a stimulus from an array of predetermined response options (Chronaki et al., 2015; Leitzke & Pollak, 2016; Pollak & Sinha, 2002). In the latter case, children are asked to either select a label to match a face (*Is this face angry*, *happy*, *or scared?*) or pick a face to match a label (*Choose the face that looks happy*). However, these approaches share three key limitations.

First, these methods are constrained by the emotion categories determined by the researcher: the researcher selects stimuli they believe represent “happy” or “sad” and accept only happy and sad as correct answers for those stimulus items. This approach can reveal the degree to which children successfully align their responses with the (adult) researcher's view of emotion (e.g., labeling a “sad” face as “sad” given the options “happy,” “angry,” and “sad”), but provide limited insight about a child's own construal of the faces, which might not map onto any of the labels or categories that the researcher selected.

Second, verbal‐response methods equate knowledge of an emotion vocabulary word with a child's use of perceptual information. This assumption can underestimate what children actually know about emotion. Many emotion words are not learned until later in development (Baron‐Cohen et al., 2010), word comprehension often precedes word production (Bergelson & Swingley, 2012, 2013), and social referencing paradigms indicate that infants are adaptively using facial movements to guide their behavior long before expressive emotion vocabulary is present (Walden & Ogan, 1988). For these reasons, it is unsound to assume that a child who cannot produce, comprehend or use a word such as “scared” does not know something about the concept of fear or threat. Furthermore, seemingly simple emotion words change in abstraction across development (Nook et al., 2020), making it difficult to interpret whether children and adults even mean the same thing when using a label such as “mad,” let alone complex ideas such as love or shame.

Third, most extant procedures were not designed to provide information about how children think about the relations among emotion cues. Past work has explored these dimensional and categorical mappings of emotion in adults (e.g., Cowen & Keltner, 2021); however, it is still unclear what these relations might look like in children, and how they develop. Some kinds of relations can be inferred through patterns of errors observed in verbal‐response paradigms—such as the consistency of children's confusion about anger versus disgust (Leitzke & Pollak, 2016; Widen & Russell, 2010b). Yet, for the most part, information about how children perceive and think about underlying relations among emotion cues is limited. This limitation also reflects a broader problem in emotion research: interpretations of children's “errors” are often predicated on the assumption that deviations from the researcher's pre‐determined label for an emotion stimulus are incorrect—that is, if the researcher has labeled a stimulus face as “sad,” other interpretations or reactions to those stimuli are coded as errors.

Here, we sought to understand how children represent emotions, without introducing verbal labels or assumptions about the accuracy of participants' responses. To do so, we adapted the spatial arrangement method (SpAM) developed by Goldstone (1994), in which participants freely sort images according to the extent to which they perceive stimuli as semantically related without imposing the use or primacy of any specific dimension, category, or label. SpAM has been used with both adults and children (Coburn et al., 2019; Hout & Goldinger, 2016; Koch et al., 2020; Richie et al., 2020; Unger et al., 2016; Vales, Stevens, et al., 2020), validated alongside more traditional pairwise similarity judgment tasks in both adults (Hout et al., 2013) and children (Unger et al., 2016), and shown to demonstrate external validity, capturing experience‐driven changes in children's semantic knowledge in domains such as plants, animals, foods, and tools (Unger & Fisher, 2019; Vales, States, et al., 2020). Furthermore, this task uses graded similarity judgments (i.e., the distance between images) to assess children's emotion knowledge, rather than labeling particular sorting strategies as right or wrong, which allows us to better characterize patterns of change across development.

We tested predictions that follow from extant theories about the emergence of human emotion, including the possibilities that (a) children use emotion categories (Izard, 2007; Keltner et al., 2019), resulting in facial configurations with the same category label being placed more closely together than those with different category labels across development; (b) children use continuous dimensions including bipolar valence and arousal (Russell, 2003), resulting in facial configurations with more similar bipolar valence and arousal ratings being placed more closely together; (c) children use valence in a bivariate manner (Larsen et al., 2009), resulting in facial configurations being placed more closely together the more similar their bivariate valence ratings; and (d) children use a combination of these aforementioned features, which predicts that the valence (bivariate and bipolar), arousal, and emotion categories will all explain unique variance in how closely children place facial cues together. It is also likely that with learning and maturation, representation of emotions changes. To explore this possibility, we tested children as young as age 3;0 (the earliest age we conjectured children may be able to use this method) through age 6;11 (when children label many emotions similarly to adults) and compared children's behaviors to those of adults. We approached the data in two distinct ways: (1) a top‐down, supervised approach to test the extent to which predefined emotion categories and dimensions predict sorting behavior, and (2) a bottom‐up, unsupervised approach examining participants’ behavior without prescribing primacy to any given theory or any specific dimension.

## METHOD

### Participants

We recruited 107 children (age range 3;0–6;11 years, *M* = 5.0, *SD* = 1.1; 48 M, 59 F; race or ethnicity: 6.5% more than one race, 84.1% White, 9.3% Asian) and 40 adults (age range: 18–21 years, *M*_age_ = 18.8, *SD* = 0.7; 10 M, 30 F; race or ethnicity: 10% Hispanic, 30% Asian, 2.5% Black, 57.5% White). Children were recruited from the community in a large Midwestern city (Madison, Wisconsin), and data were collected from June 2019 until March 2020. One 4‐year‐old child in the final sample completed only the practice sort and the Same Individual Sort, and one child was excluded because they completed only the practice sort, resulting in a final sample of 106 children (gender: 48 M, 58 F; race or ethnicity: 9.4% Asian, 84.0% White, 6.6% multiple). We aimed to have 30 children in each age bin but terminated data collection early because of the COVID‐19 outbreak; the final sample reported here includes 21 three‐year‐olds, 34 four‐year‐olds, 28 five‐year‐olds, and 23 six‐year‐olds. Twenty participants per subgroup have provided sufficient power for most cluster analysis techniques (Dalmaijer et al., 2020), and our sample size is comparable to those used in past studies using the SpAM with children (*n* = 18 per group, Unger et al., 2016).

### Stimuli

Stimuli were drawn from the Interdisciplinary Affective Science Laboratory (IASLab) Facial Stimuli Set (more information available online at https://www.affective‐science.org/face‐set.shtml). We selected actors with the highest average accuracy ratings and no facial hair. The stimuli were designated by IASLab as open and closed mouth versions of anger, calm, disgust, excitement, fear, happiness, neutral, sadness, and surprise for a total of 18 images in each sorting condition. To test for the robustness of any possible effects, each participant completed two sorting conditions. One sorting condition consisted of 18 different facial configurations posed by the same individual; the other sorting condition consisted of 18 different individuals (half male and half female, with a male and female for each emotion). In this manner, the Same Individual condition reveals how participants construe different facial configurations from one individual, whereas the Different Individual condition reflects a generalization across individual actors, allowing examination of whether similar sorting patterns emerge when a variety of different perceptual features are changing (facial cue, identity, race, and gender).

#### Ratings of stimuli

Fifty undergraduates who did not participate in the sorting task completed ratings of bipolar valence, bivariate valence (i.e., ratings of positivity and negativity), and arousal for each of the 36 images. Ratings of the stimuli were collected for use as (independent) predictors in the analyses of sorting behavior. For each image, participants completed 7‐point Likert ratings of bipolar valence and arousal (Warriner et al., 2013) and the Evaluative Space Grid for bivariate valence (ESG; Larsen et al., 2009). Valence is often treated as a bipolar measure ranging from negative at one pole to positive at the other with a neutral midpoint. However, bivariate valence—representing positivity and negativity in a two‐dimensional space—has been found to more accurately capture emotional experience (Larsen & McGraw, 2011; Watson et al., 1999). Traditional bipolar valence scales pose interpretive challenges: scores in the middle of the scale could indicate that the individual perceives the stimulus as neither positive nor negative (indifference, neutrality), that the individual perceives a mix of positivity and negativity (ambivalence, multiple emotions), or that the perceiver is uncertain (a stimulus could be either positive or negative depending upon the context). The ESG method disentangles these possibilities by presenting participants with a square depicting a 5‐point positivity scale on one axis and a 5‐point negativity scale on the other, allowing participants to select where the stimuli fall along both dimensions. Additional details on stimuli ratings are available in Supporting Information (see https://rpubs.com/zcm/GRD_supplemental).

### Design and procedure

Images were presented on a Dell 24: P2418HT touchscreen monitor using PsychoPy (version v1.83.04; Peirce et al., 2019). At the outset of each sorting condition, participants saw all the images to be sorted. The images then disappeared, and each image was presented one at a time in the center of the screen in a randomized order for each participant. Participants were able to arrange the images by touching and dragging them to any location on the grid. For the practice phase, participants were instructed to arrange the images so that “things that are of the same kind of thing go together and things that are different or not the same kind of thing go apart.” For the facial sorts, participants were told to “sort the pictures of faces based on how people might feel inside” and that “people that feel the same kind of thing go together and people that feel a different kind of thing go apart.” Participants could continue to move each image throughout the task, as all images remained viewable after they appeared. In order to ensure that images were clearly visible to participants, images would expand in size (from 140 × 140 to 315 × 315 pixels) while participants touched them to move the image, and then returned to their original size once placed in the grid. Once child participants were no longer moving any images, the experimenter asked if they were ready for the next picture. Adult participants were able to control when the next image would appear themselves by using the spacebar. Adults and children received the same task instructions, though adults were also informed at the beginning that the instructions were designed to also be appropriate for younger participants.

To introduce participants to the task, they saw four images (soccer ball, basketball, rabbit, and chair) and practiced moving them around on the screen. The grid had no labels or axes, so participants were not sorting onto a predefined space. The experiment began with a practice phase in which participants arranged five images (car, bus, squirrel, bird, table). The practice phase was not scored, because the principle of the method is that there are no wrong answers (see Supporting Information for more details); however, this phase allowed us to assess how participants approached the task independent of the emotion stimuli (by, e.g., grouping the animals together or, as one child explained, grouping the squirrel and the table together because “they both have legs”). For the next two conditions, participants saw faces and were instructed to think about how the person might be feeling, and that people feeling the same kind of thing should go together. Participants then completed a *Same Individual Condition* in which they sorted 18 facial cues of emotion for one actor (Male # 7). Next, participants completed a *Different Individual Condition*, this time sorting 18 facial configurations posed by 18 different actors (Females: # 1, 4, 7, 10, 13, 14, 15, 17, 22; Males: # 2, 3, 4, 5, 8, 12, 14, 15, 17). Full task instructions are available on Open Science Framework (OSF) (https://osf.io/7bkgp/) and in Supporting Information.

## RESULTS

Analyses were conducted in R (version 4.1.1; R Core Team, 2021), fitting linear mixed‐effects models using the lme4 package (Bates et al., 2015; version 1.1–27.1). Following the recommendations of Luke (2017), *F*‐values and *p*‐values for linear mixed‐effects models were obtained using the Satterthwaite approximation of the degrees of freedom (Kuznetsova et al., 2017). Participants’ patterns of sorting behavior were characterized by calculating the Euclidean distance between images, which were then normalized for each participant by scaling distances based on the maximum distance for each participant. All analysis code and analytic details can be found on the project's OSF page (https://osf.io/7bkgp/), including a walkthrough of each analysis (https://rpubs.com/zcm/GRD_main) and Supporting Information (https://rpubs.com/zcm/GRD_supplemental). We first conducted a series of analyses using top‐down, supervised approaches, followed by a series of analyses using bottom‐up, unsupervised approaches. The analyses conducted were exploratory in nature, implementing similar approaches to those applied in past studies using the SpAM (Unger et al., 2016); however, converging patterns of results across multiple different analyses give us increased confidence in the robustness of the results. Analysis of the practice phase is in Section 1 of the Supporting Information.

### Dimensions of affect and categories in sorting behaviors

We began with top‐down, supervised methods to examine whether emotion category and dimensions of affect account for how closely different facial cues are placed to one another. We examined these features separately, and then compare how well the various dimensions and categories account for sorting behaviors.

#### Emotion category

We first investigated developmental change in the use of common English language emotion categories (e.g., sad, happy, anger, disgust, fear, surprise, neutral, calm, excitement) as a structure for emotion cues. To do so, we computed the average distance between images that shared the same category label (e.g., the distance of one happy face to another happy face) versus images that had differing category labels (e.g., the distance of one happy face to a sad face) for each participant (see also Unger et al., 2016 for a similar approach). To do so, we fit a linear mixed‐effects model estimating the average distance between item pairs for adults versus children (coded 0.5, −0.5), the category match for an image pair (same category pair vs. different category pair; centered: same = 0.5, different = −0.5), and their interaction with a by‐participant random intercept and a by‐participant random slope for category match. We analyzed results collapsing across sorting conditions, as there was no evidence that results differed between the same and different individual sorts (*p* = .25). Adults were more likely than children to place images belonging to the same emotion categories closer together than images belonging to different emotion categories, *b* = −.15, Wald 95% CI [−.18, −.12], *F*(1, 172.86) = 125.42, *p* < .001. We find the same pattern of results if only the most basic emotion categories (happiness, sadness, anger, disgust, fear, and surprise) are included in the analysis (see Section 2 of Supporting Information).

**FIGURE 1:**
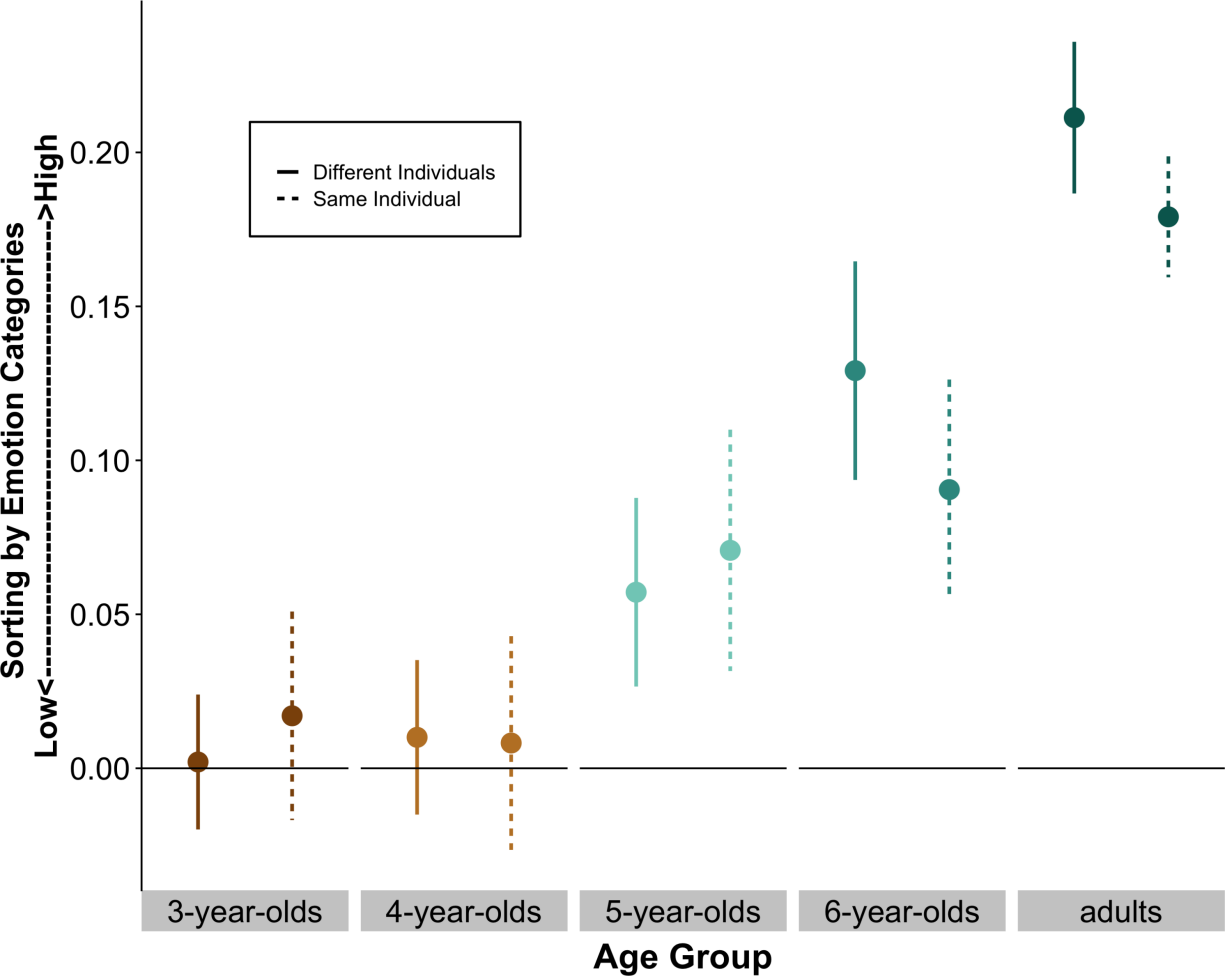
Use of emotion categories in sorting behavior. *Y*‐axis represents the difference in average distance for items belonging to the same versus different emotion categories. An average value of zero represents no distinction by emotion category, as faces from the same versus different emotion categories were equally far apart. Error bars represent 95% confidence intervals

To understand how children's use of emotion categories changed across development, we next fit a linear mixed‐effects model on the child data with age (in years; centered) as a continuous predictor with an otherwise identical model structure. Children were more likely to sort facial configurations based upon emotion category labels with increasing age, *b* = −.03, Wald 95% CI [−.05, −.02], *F*(1, 130.91) = 28.13, *p* < .001. This developmental increase in use of category labels is shown in Figure 1 (see Section 2 in Supporting Information for plots representing age as a continuous variable). Follow‐up analyses of each age group separately reveals that neither 3‐year‐olds (*p* = .45) nor 4‐year‐olds (*p* = .47) showed evidence of sorting based upon emotion categories, while 5‐year‐olds (*b* = −.06, Wald 95% CI [−.09, −.04], *F*(1, 31.81) = 22.27, *p* < .001) and 6‐year‐olds (*b* = −.11, Wald 95% CI [−.14, −.08], *F*(1, 33.94) = 65.47, *p* < .001) began using category information, though to a lesser extent than adults (*b* = −.20, Wald 95% CI [−.21, −.18], *F*(1, 76.20) = 490.88, *p* < .001).

#### Dimensions of affect

Next, we tested whether bipolar valence, bivariate valence (separate ratings of positivity and negativity), and arousal predicted participants’ sorting behavior. To do so, we fit a series of linear mixed‐effects models regressing the average distance between item pairs on their similarity along the dimension of interest (bipolar valence, arousal, positivity, and negativity)—measured in terms of the difference in average stimulus rating between image pairs. This analysis included age group (adults: 0.5; children: −0.5), its interaction with the dimension of interest, and random effects for items and participants, including a by‐participant random intercept, a by‐participant random slope for the dimension of interest, and a by‐item‐pair random intercept. Adults were more likely than children to use each of the four dimensions to guide their sorting behaviors (bipolar valence: *b* = .07, Wald 95% CI [.06, .08], *F*(1, 143.84) = 147.96, *p* < .001; arousal: *b* = .03, Wald 95% CI [.02, .04], *F*(1, 143.63) = 43.30, *p* < .001; positivity: *b* = .10, Wald 95% CI [.08, .11], *F*(1, 143.86) = 146.17, *p* < .001; negativity: *b* = .11, Wald 95% CI [.09, .13], *F*(1, 143.73) = 111.52, *p* < .001). To further understand the developmental change in children's use of each dimension, we fit linear mixed‐effects models on the child data with age (in years; centered) as a continuous predictor and an otherwise identical model structure. Children increasingly used each feature across development (valence: *b* = .01, Wald 95% CI [.01, .02], *F*(1, 103.89) = 31.08, *p* < .001; positivity: *b* = .01, Wald 95% CI [.01, .02], *F*(1, 103.88) = 21.32, *p* < .001; negativity: *b* = .03, Wald 95% CI [.02, .03], *F*(1, 103.81) = 35.36, *p* < .001)—with the exception of arousal, *b* = .003, Wald 95% CI [−.001, .01], *F*(1, 103.81) = 1.97, *p* = .16. The pattern for arousal highlights how children's development may not always occur as straightforward linear differentiation (see Section 3 of Supporting Information for additional details).

#### Comparing dimensions of affect and emotion categories in sorting behaviors

Finally, we examined how well emotion category predicted participant's sorting behavior compared to valence and arousal. To do so, we computed the average distance between all stimulus pairs (*n* = 306 unique pairs) for each age group and predicted these distances from a pair's similarity on each dimension of interest simultaneously. This general linear model revealed how much each dimension aided in explaining variance in each age group's sorting behavior. First, we estimated the use of bipolar valence, arousal, and whether image pairs shared the same discrete emotion category (0 = different category pair; 1 = same category pair). Second, we estimated the effects of bivariate valence with positivity and negativity as two orthogonal dimensions.

##### Bipolar valence, arousal, and shared emotion category

**FIGURE 2:**
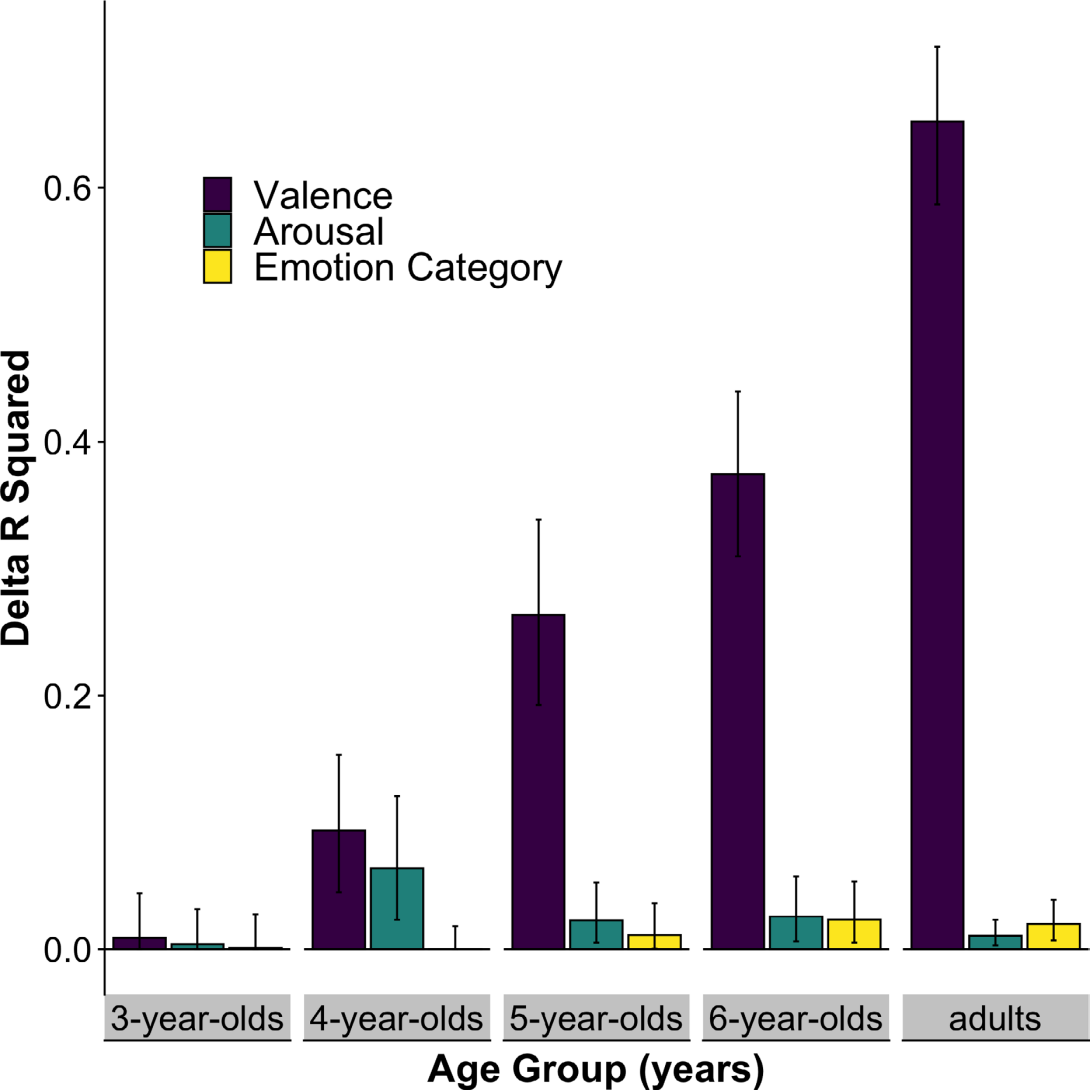
Delta *R* ^2^ for each predictor of sorting behavior. Error bars represent bootstrapped 95% confidence intervals

Valence emerged as (by far) the strongest predictor (Figure 2) of how participants grouped facial images, an effect that increased steadily with age. Arousal was a significant predictor for 4‐year‐olds, but declined as children grew older. Consistent with the results from the previous section, emotion category did not emerge as a predictor until age 5 years. The total variance explained by this model increased steadily across age (Table 1), accounting for a significant amount of the error variance for all age groups (*F*(3, 302) > 14, *p* < .001) with the exception of the youngest age group (3‐year‐olds: *F*(3, 302) = 1.33, *p* = .26).

**TABLE 1:** Predicting sorting distance from valence, arousal, and shared emotion category

Predictor	Estimate	*t*‐value	*p*	Δ*R* ^2^	Overall *R* ^2^
3‐year‐olds					.01
Valence	0.004	1.67	.10	.01	
Arousal	−0.004	−1.12	.27	.00	
Emotion category	−0.006	−0.55	.58	.00	
4‐year‐olds					.13
Valence***	0.01	5.71	<.001	.09	
Arousal***	−0.02	−4.71	<.001	.06	
Emotion category	−0.001	−0.15	.88	.00	
5‐year‐olds					.31
Valence***	0.03	10.76	<.001	.26	
Arousal**	−0.02	−3.16	.002	.02	
Emotion category*	−0.03	−2.22	.027	.01	
6‐year‐olds					.46
Valence***	0.05	14.49	<.001	.37	
Arousal***	−0.02	−3.82	<.001	.03	
Emotion category***	−0.06	−3.63	<.001	.02	
Adults					.78
Valence***	0.09	30.04	<.001	.65	
Arousal***	−0.02	−3.83	<.001	.01	
Emotion category***	−0.08	−5.26	<.001	.02	

* *p* < .05;

** *p* < .01;

*** *p* < .001.

##### Bivariate valence, arousal, and shared emotion category

We repeated the previous analysis, replacing bipolar valence with bivariate valence (positivity and negativity as independent predictors). As expected, ratings of positivity and negativity were highly correlated with bipolar ratings, precluding us from including all five predictors in the same model. The dimension of negativity emerged as the strongest predictor of sorting behavior across all age ranges, even 3‐year‐olds, and explained substantially more unique variance than positivity, arousal, and emotion category (Table 2).

**TABLE 2:** Predicting sorting distance from positivity, negativity, arousal, and shared emotion category

Predictor	Estimate	*t*‐value	*p*	Δ*R* ^2^	Overall *R* ^2^
3‐year‐olds					.02
Positivity	−0.005	−1.01	.31	.00	
Negativity*	0.01	2.23	.027	.02	
Arousal	−0.00004	−0.01	.99	.00	
Emotion category	−0.004	−0.33	.74	.00	
4‐year‐olds					.18
Positivity	−0.003	−0.66	.51	.00	
Negativity***	0.02	5.60	<.001	.09	
Arousal*	−0.009	−2.42	.02	.02	
Emotion category	0.004	0.42	.67	.00	
5‐year‐olds					.45
Positivity*	−0.01	−2.48	.014	.01	
Negativity***	0.07	12.02	<.001	.26	
Arousal	0.006	1.22	.22	.00	
Emotion category	−0.02	−1.31	.19	.00	
6‐year‐olds					.57
Positivity	−0.01	−1.27	.21	.00	
Negativity***	0.09	13.87	<.001	.27	
Arousal	0.01	0.92	.36	.00	
Emotion category**	−0.04	−2.89	.004	.01	
Adults					.80
Positivity***	0.05	8.36	<.001	.05	
Negativity***	0.11	15.39	<.001	.16	
Arousal	−0.003	−0.54	.59	.00	
Emotion category***	−0.08	−4.92	<.001	.02	

* *p* < .05;

** *p* < .01;

*** *p* < .001.

##### Does bivariate valence predict sorting behavior better than bipolar valence?

To determine whether separate dimensions of positivity and negativity were better predictors than bipolar valence, we compared the models including bipolar valence to the models including positivity and negativity (bivariate valence) in each age group. Bivariate dimensions of valence were a better predictor of sorting behavior in all but the youngest age group, with the most substantial gains among the 5‐ and 6‐year‐olds (3‐year‐olds: *F*(1, 301) = 2.82, *p* = .09; 4‐year‐olds: *F*(1, 301) = 18.98, *p* < .001; 5‐year‐olds: *F*(1, 301) = 74.58, *p* < .001; 6‐year‐olds: *F*(1, 301) = 77.51, *p* < .001; adults: *F*(1, 301) = 21.88, *p* < .001).

### Bottom‐up assessment of facial cues of emotion

Next, we conducted a series of analyses using unsupervised methods to provide a complementary perspective on how emotions might be represented. We considered Same and Individual Sorts separately because the following analyses require pairwise distances between all items, which are only available within a given sorting block. The unsupervised analyses extract patterns from the sorting data by using the pairwise distances between all of the stimuli without regard to the labels or affective ratings of those stimuli. This allows us to represent differences in how children and adults are approaching the task without relying on any predetermined dimensions or categories. In order to facilitate comparisons between all of the analyses in the paper, we also investigate the extent to which sorting patterns extracted in the unsupervised analyses can be predicted from emotion category labels and affective dimensions.

**FIGURE 3:**
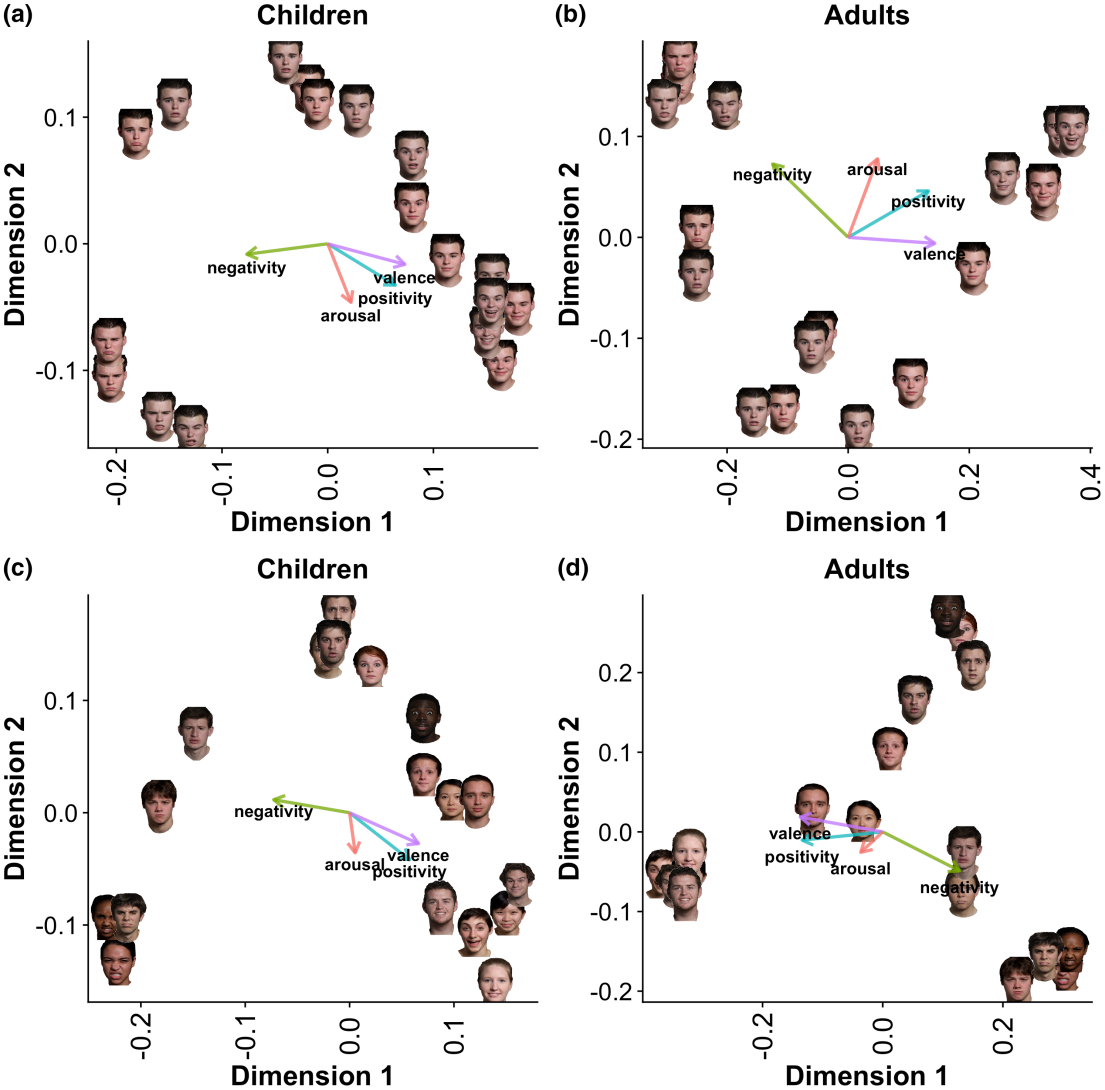
Classical multidimensional scaling solutions for all children and adults. Each panel shows the classical multidimensional scaling solution (2 dimensions) for average sorting distances across all children and adults in the Same Individual (a, b) and Different Individual (c, d) Sorts. Vectors show squared correlation coefficients between image ratings and the multidimensional scaling dimensions

First, we used two‐dimensional multidimensional scaling (MDS) to visually represent participants’ classifications (Figure 3). To better understand the underlying dimensions, we fit vectors of image ratings for bipolar valence, arousal, positivity, and negativity onto our MDS solution over 1000 permutations to derive the squared correlation coefficient of each vector (envfit in the R package vegan; Oksanen, 2019). This analysis reveals that stimuli ratings of valence, positivity, and negativity consistently correlate with the MDS dimensions (*r* ^2^ > .84 and *p* < .001 across all sort conditions for both adults and children). Arousal only correlated with the dimensions in the Same Individual Sort (adults: *r* ^2^ = .40, *p* < .05; children: *r* ^2^ = .43, *p* < .05) but not in the Different Individual Sort (adults: *r* ^2^ = .10, *p* = .46; children: *r* ^2^ = .22, *p* = .16). Additional MDS visualizations are provided in Section 5 of the Supporting Information.

**FIGURE 4:**
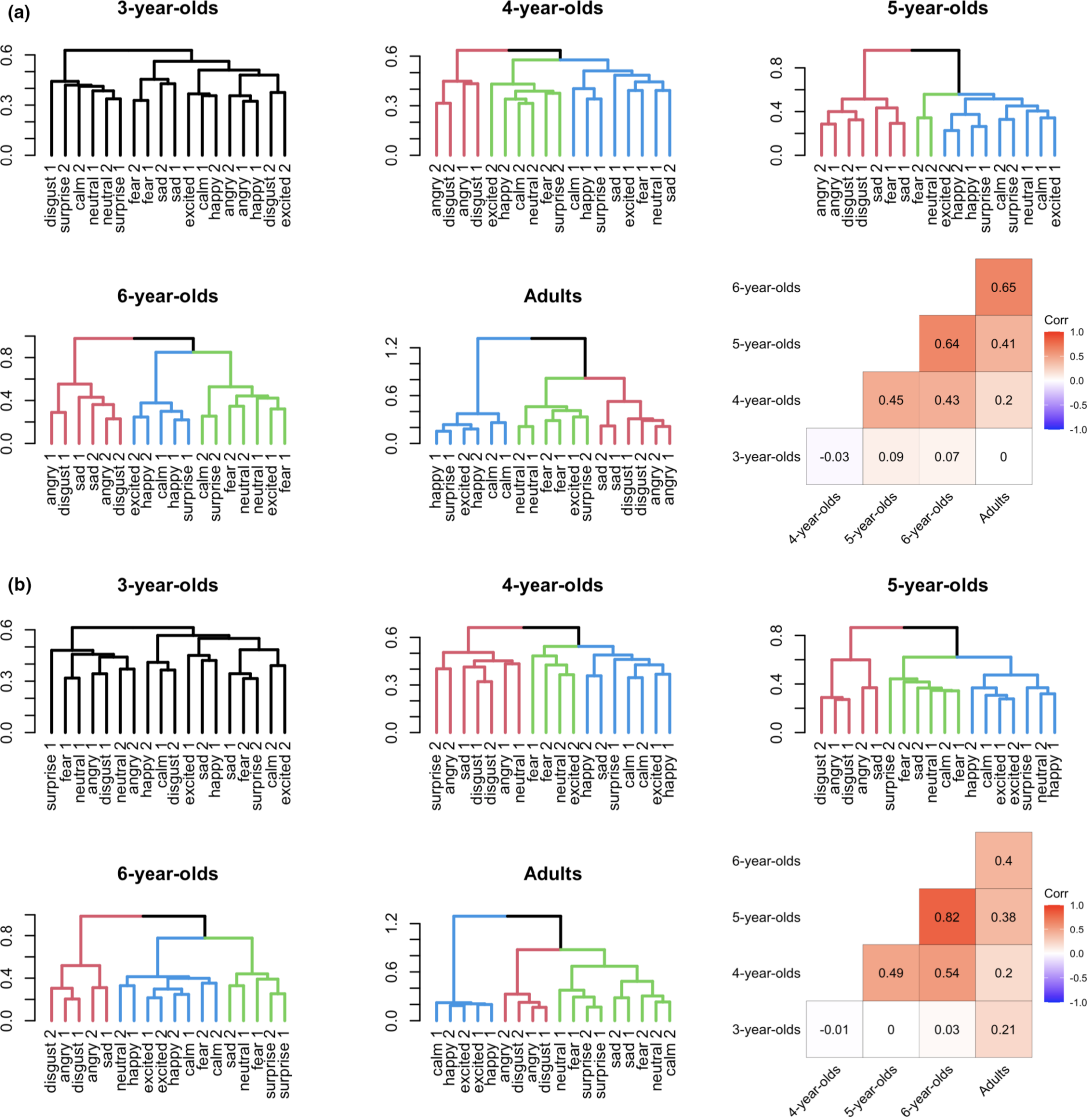
Dendrograms and correlations between dendrogram structures for the (a) same individual sort and (b) different individual sort. The numbers 1 and 2 indicate that the images had open and closed mouths, respectively. Colors specify the three cluster solutions for each age bin and highlight commonalities across dendrograms. Red clusters contain mainly anger and disgust images, green clusters mainly contain certain fear and neutral images, and blue images mainly contain certain happy, calm, and surprise images. 3‐year‐olds’ dendrograms are colored differently as they showed less differentiation

Second, we used hierarchical clustering to examine age‐related changes in how participants organized emotion cues (Ward's method; Ward, 1963), as in prior work with children and SpAM (e.g., Unger et al., 2016; Vales, Stevens, et al., 2020). This analysis allowed us to examine similarities in how adults and children sorted the facial stimuli, without using adult emotion categories or affective ratings to represent similarity between stimuli. Clustering was performed on distance matrices calculated for each age group in each sorting condition using the pairwise distances between all sorted images (see Section 6 in the Supporting Information for further details). As expected, children's clustering structures become increasingly adult‐like (Table 3; Figure 4). Children's increasingly adult‐like structures appear to be driven by changes in emotion knowledge and not improvement on the task generally, as the practice structure is highly similar to adults for all age groups except 3‐year‐olds. Given that 3‐year‐olds demonstrate relatively little consistency in their sorting strategies during the practice phase, we recommend caution in the interpretation of their results (see Section 1 in the Supporting Information for further discussion). Changes in children's clusters otherwise show strong evidence of systematicity, as children closer in age are more similar to one another. For example, the sorting behavior of 5‐year‐olds had a stronger correlation with 6‐year‐olds and 4‐year‐olds than with adults.

**TABLE 3:** Comparison of children's hierarchical clustering solutions to adult's clustering solutions

Age Group	Practice sort		Same individual sort		Different individuals sort	
	Adj. Rand (*k* = 3)	*c*	Adj. Rand (*k* = 3)	*c*	Adj. Rand (*k* = 3)	*c*
3‐year‐olds	0.21	−0.03	0.02	0	0.16	0.21
4‐year‐olds	1.0	0.86	0.14	0.2	0.14	0.2
5‐year‐olds	1.0	0.99	0.49	0.41	0.49	0.38
6‐year‐olds	1.0	0.98	0.83	0.65	0.38	0.40

Note

Each value in the table represents the similarity between children's clustering at a specific age group and adults’ clustering solution. An adjusted Rand index of 0 indicates two clusters have a Rand index that matches the expected value for random groupings, with higher and lower values indicating higher‐ or lower‐than‐chance‐level similarity between the two clusters. *c* is the cophenetic correlation coefficient between the two dendrograms. This value ranges from −1 to 1 with values near 0 suggesting that the two dendrograms are not statistically similar. Additional measurements of similarity for all values of *k* are available in Section 6 of the Supporting Information.

These changes in organizational structure can also be seen in dendrograms, which visualize the hierarchical clustering solutions. Each facial image is a node on the dendrogram that forms another node (represented by a horizontal line) when it merges with another face. Clusters are determined by the vertical height of the branches in a dendrogram, not by which labels are closest to one another laterally. Thus, faces that were found to be the most similar would be connected as a node with a very low height. To ensure that the hierarchical clustering solutions captured meaningful groupings of emotion cues, we predicted the bipolar valence ratings of cues using cluster group (*k* = 3). As in the distance‐based analyses above, bipolar valence was a strong predictor of both children's (Same Individual Sort: *F*(2, 15) = 73.86, *p* < .001; Different Individual Sort: *F*(2, 15) = 26.03, *p* < .001), and adults’ (Same Individual Sort: *F*(2, 15) = 51.87, *p* < .001; Different Individual Sort: *F*(2, 15) = 61.71, *p* < .001) cluster groups.

## DISCUSSION

This study reveals developmental changes in how humans represent perceptual information associated with emotions. By using a non‐verbal, open‐ended procedure, we circumvented a number of traditional limitations incurred in the assessment of emotion knowledge in young children. We found that children primarily rely upon the affective dimension of valence. Adult‐like reliance on common English language emotion categories (happy, sad, angry, etc.) emerged only gradually, with little evidence that children consistently used these categories until around 5 years of age. Similar patterns of incremental change in how children represent emotion emerged in both supervised and unsupervised analyses.

### Nuances in the use of valence

Valence accounted for a very large proportion of all participants' emotion judgments, providing converging evidence with prior studies (e.g., Jackson et al., 2019; Nook et al., 2017). Even 3‐year‐olds used negativity to guide their sorting behaviors, although this result should be interpreted cautiously given how this age group approached the practice sort (see Section 1 of Supporting Information). Our findings of an early role for negativity in children's emerging emotion knowledge is consistent with reports that young children display greater knowledge of negative emotions (Lagattuta & Wellman, 2001), attend more to negative faces (Lagattuta & Kramer, 2017), engage in greater discussion of negative emotions (Lagattuta & Wellman, 2002), and voluntarily explore negatively valenced stimuli (Grisanzio et al., 2021).

The present data also reveal new insights about valence. First, treating valence as bivariate (represented by separate unipolar scales of positivity and negativity) better accounted for behavior than treating it as a bipolar construct (a single continuum ranging from positive to negative). Second, positivity and negativity are not used equally early in development. Young children relied heavily on negativity and did not consistently use positivity. Allowing positivity and negativity to exist separately might also better capture human experience: One can experience spicy food as both painful and delicious, or horror movies as both frightening and entertaining (although see Russell, 2017 for a critique on how bivariate valence may play a role in judgments about affect but not experienced affect).

### The limits of arousal

Though often discussed in tandem with valence, we found that arousal decreased in use across development after age four, explained a much smaller proportion of behavior than valence, and did not consistently correlate with any MDS solution. This limited role of arousal is in contrast with many theories of emotion that posit that emotions initially emerge from a 2‐factor understanding of valence and arousal (for reviews see Barrett & Bliss‐Moreau, 2009; Russell & Barrett, 1999). There are a number of reasons for these divergent conclusions. First, arousal can be presented to research participants in different ways—such as perceptions of excitement, activation, or intensity in the self or others—that elicit varying interpretations. Second, arousal is sometimes offered as a speculative explanation of the data without measuring arousal using independent ratings (e.g., Bliss‐Moreau et al., 2020; Nook et al., 2017). Third, arousal may index natural covariation in positivity and negativity, rather than capturing unique variance in emotion (Haj‐Ali et al., 2020; Kron et al., 2013). Our data support this third possibility, as the variance explained by arousal largely disappears when we use bivariate valence (see Table 2).

### What changes in the structure of emotion knowledge over development?

Our data suggest that developmental changes in how emotions are represented do not simply reflect children's responses becoming more consistent or children becoming more competent at the task with increasing age; instead, the manner in which children prioritized and used different dimensions of emotion changed across age. With the exception of the 3‐year‐old age group, children demonstrated good comprehension of the task during the practice phase and sorted items unrelated to emotion similarly to adults. Starting at 4 years of age, children systematically organized facial configurations according to broader dimensions, with some dimensions (e.g., valence) gaining increasing explanatory weight and other, initially influential dimensions (e.g., arousal) diminishing in effect size with age. Moreover, children's sorting patterns were distinctive: Children closer in age had clustering structures that were much more aligned with one another than with those of adults. These results suggest that children prioritize perceptual information about emotion in a systematic manner that is distinct from how adults organize this same information.

The patterns that we observed in the development of emotion knowledge appear similar to those discovered in other domains of development. For instance, the development of non‐emotional categories (e.g., animals and other natural kinds) reveals that children first make broad distinctions (e.g., animals vs. tools) and later show finer differentiation of items based on their category membership (e.g., birds vs. mammals; Vales, Stevens, et al., 2020). The present data uncover a similar pattern of finer‐grained differentiation across development for emotion knowledge. We found that children first use broad, primarily valence‐based distinctions, and with greater experience, draw more fine‐grained distinctions that use emotion category information (Matthews et al., 2020; Widen, 2013). Rather than a distinct shift from using valence to using emotion categories, we found continued and refined use of valence and emotion categories across development. These findings contradict some infant research, which finds that discrete emotion categories emerge earlier than superordinate categories like valence (Ruba et al., 2017, 2020; White et al., 2019). However, this discrepancy may be due to methodological differences, as infant research focuses more on perceptual discrimination (for a full discussion of this issue, see Ruba & Pollak, 2020) rather than graded similarity judgments. Furthermore, the present study allows children to use both valence and discrete categories at the same time (rather than having the two sources of information compete). While valence and discrete categories are often pitted against one another, we found that the two are often related. For instance, anger and disgust had the most negative valence ratings, while happy faces tended to have some of the most positive valence ratings. Thus, knowledge of valence can often give a learner traction on knowledge that appears to be category‐related, and vice versa. We find increased use of both valence information and category information across development in the current data.

The changes we observed in children's behavior could also reflect transitions in conceptual development. Children may shift from more perceptual, similarity‐based categories to categories shaped more by rules and labels (see Sloutsky & Deng, 2019). A related possibility is that growth of emotion vocabulary, including more abstract conceptions of emotion, gradually reshape children's representations of emotion (Hoemann et al., 2020; Nook et al., 2020).

### Limitations and future directions

We attempted to introduce some degree of variation and diversity into our stimuli by including open and closed mouthed images of nine emotion categories, from Asian, Black, and White males and females. But a fuller understanding of emotional development will require even greater variety in (a) the age, gender, ethnic, and racial identities of the individuals providing emotion cues; (b) use of emotion categories beyond those commonly used in English; (c) stimuli that are naturally occurring rather than posed; (d) less reliance on faces alone and more emphasis on the situational contexts and broader variety of dynamic visual and auditory stimuli that characterize human interactions (Srinivasan & Martinez, 2018; Woodard et al., 2021). Future research might also explore how children construe experimental tasks such as the one we used here. One advantage of the current task is that children are given minimal verbal prompting to guide their sorting behavior, allowing us to study children's spontaneous emotion judgments. However, research on conceptual development reveals that even subtle variations in task context, such as the verbal prompts used to introduce the task, can reveal different facets of children's knowledge (e.g., Christie & Gentner, 2014; Deák & Bauer, 1995; Waxman & Namy, 1997). For instance, future studies could ask children to consider how others might act next (given the presence of emotion‐relevant cues), frame the task using specific emotion labels, or present the task under different contexts. The consistency in use of information across all of these different framings would lend strong support to task‐independent representations of perceptual information about emotion, while variability would provide evidence for context‐sensitive use of different factors when children evaluate emotion cues.

## CONCLUSIONS

Emotions are critical for human adaptation and survival, yet relatively little is understood about how humans come to understand and represent emotion signals. Several explanations commonly used to account for the emergence of emotion find little or only partial support in the present data. Young children in our task did not begin to use basic emotion categories until around the age of 5, arguing against the theory that this knowledge plays a large role in young children's emotion understanding. Children also did not rely equally on the dimensions of valence and arousal, instead using negative valence far more heavily. The picture of emotion development that emerges from our data is of an incremental learning process in which children change their representations of emotion using combinations of factors that are weighted differently across development. This insight opens the door for new investigations about how humans learn to navigate the complex communicative system of the social world.

## Supporting information

Supinfo
