# Supplementary material for: The representation of emotion knowledge across development
Source: Child Dev. 2021 Nov 25;93(3):e237–50. doi: 10.1111/cdev.13716 (PMC9203044; doi:10.1111/cdev.13716)
Supplement: Supplementary file 1 — Supinfo [file CDEV-93-e237-s001.html]

Supplemental Materials


Code 

- Show All Code
- Hide All Code

# Supplemental Materials

#### Kristina Woodard, Martin Zettersten, & Seth Pollak

#### 21 November, 2021

This document contains supplemental materials for the paper:

Woodard, K., Zettersten, M., & Pollak, S.D. (accepted). The representation of emotion knowledge across development. Child Development.

See the OSF site for the paper for further information:

https://osf.io/7bkgp/

# 1. Practice Phase

To assess whether participants in each age group demonstrated understanding of the grid task during the practice phase, we investigated the degree to which participants consistently arranged images belonging to the same superordinate categories (vehicles: car and bus; animals: squirrel and bird) closer together in space. Below is an example of what a practice sort might look like:

We computed the average distance between images belonging to the same superordinate category (vehicles or animals) for each participant, and then compared the average distances for item pairs sharing the same category to item pairs from different categories. 3-year-olds did not consistently arrange items belonging to the same superordinate category closer together in space, (paired t-test: t(20) = -0.046, p = .96). This suggests that children in this age group were not consistently sorting images according to superordinate categories and may have struggled with the task instructions. However, we included 3-year-olds in all analyses as we had no reason to exclude this group a priori. All other age groups consistently sorted images belonging to the same category closer together in the grid space (4-year-olds: t(34) = 5.03, p < .001; 5-year-olds: t(27) = 5.42, p < .001; 6-year-olds: t(22) = 8.55, p < .001; adults: t(39) = 29.10, p < .001).

3-year-olds during the practice phase did not appear to use superordinate categories to guide their similarity judgments. Rather, children in this age range appeared to approach the task with many different strategies: some did appear to use superordinate categories like animals and vehicles (n = 5), some separated each image far apart, viewing each stimulus as distinct (n = 4), some appeared to focus on other perceptual features, like color, to guide their decisions (n = 5), and some appeared to sort at random (n = 7). However, our observations in this regard are subjective and future research will need to adjust the task to better understand and tease apart possibilities for how these youngest children understood the stimuli.

```
library(broom)
library(gt)
# get average dist based on category_pair and age bin
avg_dist_cat_by_subj <- subj_dist_long %>%
  group_by(subject, Age, Gender, age_bin,age_group,sort,category_pair) %>%
  summarize(N=n(),avg_dist=mean(dist),ci_dist=qt(0.975, N-1)*sd(dist,na.rm=T)/sqrt(N))

#paired  t-test on average distance scores
#3 year olds
t1 <- t.test(filter(avg_dist_cat_by_subj,age_bin=="3 to 4"& sort=="Practice"&category_pair=="between")$avg_dist,
       filter(avg_dist_cat_by_subj,age_bin=="3 to 4"& sort=="Practice"&category_pair=="within")$avg_dist,
       paired=T)
#4 year olds
t2 <-t.test(filter(avg_dist_cat_by_subj,age_bin=="4 to 5"& sort=="Practice"&category_pair=="between")$avg_dist,
       filter(avg_dist_cat_by_subj,age_bin=="4 to 5"& sort=="Practice"&category_pair=="within")$avg_dist,
       paired=T)
#5 year olds
t3 <-t.test(filter(avg_dist_cat_by_subj,age_bin=="5 to 6"& sort=="Practice"&category_pair=="between")$avg_dist,
       filter(avg_dist_cat_by_subj,age_bin=="5 to 6"& sort=="Practice"&category_pair=="within")$avg_dist,
       paired=T)
#6 year olds
t4 <-t.test(filter(avg_dist_cat_by_subj,age_bin=="6 to 7"& sort=="Practice"&category_pair=="between")$avg_dist,
       filter(avg_dist_cat_by_subj,age_bin=="6 to 7"& sort=="Practice"&category_pair=="within")$avg_dist,
       paired=T)
#adults
t5 <-t.test(filter(avg_dist_cat_by_subj,age_bin=="adults"& sort=="Practice"&category_pair=="between")$avg_dist,
       filter(avg_dist_cat_by_subj,age_bin=="adults"& sort=="Practice"&category_pair=="within")$avg_dist,
       paired=T)

tab <- map_df(list(t1, t2, t3, t4, t5), tidy)
tab$age = c("3-year-olds","4-year-olds","5-year-olds","6-year-olds","adults")
tab <- tab[c("age","estimate","statistic","p.value")]

tab1<- gt(tab) %>% cols_align(align="center") %>% cols_label(age = md("**Age Groups**"), estimate=md("**Estimate**"), statistic=md("**Statistic**"), p.value=md("**P-value**")) %>%   tab_header(
    title = md("Practice Phase T-tests"))
tab1
```

| Practice Phase T-tests | | | |
| --- | --- | --- | --- |
| **Age Groups** | **Estimate** | **Statistic** | **P-value** |
| --- | --- | --- | --- |
| 3-year-olds | -0.002234998 | -0.04561796 | 9.640672e-01 |
| 4-year-olds | 0.191338142 | 5.03164582 | 1.680473e-05 |
| 5-year-olds | 0.279394461 | 5.42221631 | 9.816049e-06 |
| 6-year-olds | 0.394844175 | 8.55117648 | 1.919296e-08 |
| adults | 0.493390050 | 29.09477256 | 4.660640e-28 |

# 2. Sorting by Emotion Categories

## Continuous Age Predictor

In the manuscript we examined whether images belonging to the same or different emotion categories were more likely to be placed near one another. Age was binned into 4 age groups. In the following analysis, we show sorting by emotion category across child age (as a continuous predictor) with a LOESS regression fit. We see a qualitative shift around 5 years of age in sorting by emotion category.

```
avg_dist_diff_cat_by_subj <- subj_dist_long %>%
  group_by(subject, Age, Gender, age_bin,age_group,sort,category_pair) %>%
  summarize(avg_dist=mean(dist,na.rm=T))  %>%
  ungroup() %>%
  group_by(subject, Age, Gender, age_bin,age_group,sort) %>%
  pivot_wider(names_from=category_pair,values_from=avg_dist) %>%
  mutate(avg_dist_diff=between-within) %>%
  mutate(sort_name=ifelse(sort=="Sort1","Same Individual",ifelse(sort=="Sort2","Different Individuals","Practice"))) %>%
  ungroup()

ggplot(filter(avg_dist_diff_cat_by_subj,age_group=="kids"&sort!="Practice"),aes(Age,avg_dist_diff))+
  geom_point()+
  geom_smooth(size=2)+
  geom_hline(yintercept=0)+
  facet_wrap(~sort_name)+
  ylab("Sorting by Emotion Categories \nLow<------------------------>High")+
  xlab("Age (in years)")
```

## Basic Emotions

In the manuscript we examined whether images belonging to the same or different emotion categories were more likely to be placed near one another. However, since some individuals might object to including emotion categories like “neutral” or “calm”, we ran an additional analysis where we only included basic emotions: anger, disgust, fear, happiness, sadness, and surprise. We found the same pattern of results as when all emotion categories are included.

```
# Plot
p <- ggplot(filter(avg_dist_diff_cat_across_subj, sort!="Practice"),aes(x=sort_name,y=average_dist,fill=age_bin,color=age_bin, linetype=sort_name))+
  geom_errorbar(aes(ymin=average_dist-ci_dist,ymax=average_dist+ci_dist),width=0,size=1)+
  geom_point(size=2.5)+
  facet_wrap(~age_bin,nrow=1, labeller = age_labeller, strip.position = c("bottom"))+
  geom_hline(yintercept=0)+
  theme(legend.position=c(.1,.8), legend.title = element_blank(),
        legend.margin = margin(.5,.5,.5,.5,"cm"),
        legend.background = element_rect(fill="white",size=0.6,
                                         linetype="solid", colour ="black")) +
  scale_color_manual(values=c("#8c510a","#bf812d","#80cdc1","#35978f","#01665e"))+
  guides(color=FALSE) +
  guides(fill=FALSE) +
  ylab("Sorting by Emotion Categories \nLow<------------------------>High")+
  xlab("Age Group")+
  ylim(-.1,.3) +
  labs(linetype = "Sorting Phase") +
  theme(axis.line = element_blank(),
    axis.text.x  = element_blank(),
        axis.ticks.x= element_blank(),
        axis.title.x = element_text(size=20,face="bold"),
        axis.text.y =  element_text(size=18),
        axis.title.y= element_text(size=18,face="bold"),
        strip.text.x = element_text(size=18)) #+
p
```

## Emotion Category

In the manuscript we examined whether images belonging to the same or different emotion categories were more likely to be placed near one another. Here we show this analysis in more detail by showing the effect for each individual emotion category, rather than collapsing across all categories. Overall, we see similar effects across each emotion category.

```
p <- ggplot(avg_dist_diff_cat_across_subj,aes(x=age_bin,y=average_dist,fill=age_bin,color=age_bin, linetype=sort_name))+
  geom_errorbar(aes(ymin=average_dist-ci_dist,ymax=average_dist+ci_dist),width=0,size=1, position=position_dodge(width=0.5))+
  geom_point(size=1.5, position=position_dodge(width=0.5))+
  facet_wrap(~shared_category_labels, nrow=3, strip.position = c("top"))+
  geom_hline(yintercept=0)+
  scale_color_manual(values=c("#8c510a","#bf812d","#80cdc1","#35978f","#01665e"))+
  ylab("Sorting by Emotion Category \nLow<------------------------>High")+
  xlab("Age Group")+
  labs(linetype = "Sorting Phase:", color = "Age:", fill = "Age:") +
  theme(legend.position="top", legend.box = "vertical", legend.justification = "center") +
  theme(axis.line = element_blank(),
    axis.text.x  = element_blank(),
        axis.ticks.x= element_blank(),
        axis.title.x = element_text(size=20,face="bold"),
        axis.text.y =  element_text(size=18),
        axis.title.y= element_text(size=18,face="bold"),
        strip.text.x = element_text(size=18))
p
```

## Evaluative Space Grid Categories

The Evaluative Space Grid (ESG) is often divided into four sections: polarizing positive (high positivity, low negativity), polarizing negative (high negativity, low positivity), indifferent (low positivity and negativity), and ambiguous (high positivity and negativity). Here we examine if images belonging to the same ESG categories are placed more closely together. Notably, we see that by around 4-year-of-age children are using ESG category to guide their sorting behaviors.

```
p <- ggplot(filter(avg_dist_diff_esg_across_subj, sort!="Practice"),aes(x=sort_name,y=average_dist,fill=age_bin,color=age_bin, linetype=sort_name))+
  geom_errorbar(aes(ymin=average_dist-ci_dist,ymax=average_dist+ci_dist),width=0,size=1)+
  geom_point(size=2.5)+
  facet_wrap(~age_bin,nrow=1, labeller = age_labeller, strip.position = c("bottom"))+
  geom_hline(yintercept=0)+
  theme(legend.position=c(.1,.8), legend.title = element_blank(),
        legend.margin = margin(.5,.5,.5,.5,"cm"),
        legend.background = element_rect(fill="white",size=0.6,
                                         linetype="solid", colour ="black")) +
  scale_color_manual(values=c("#8c510a","#bf812d","#80cdc1","#35978f","#01665e"))+
  guides(color=FALSE) +
  guides(fill=FALSE) +
  ylab("Sorting by ESG Category \nLow<------------------------>High")+
  xlab("Age Group")+
  ylim(-.1,.3) +
  labs(linetype = "Sorting Phase") +
  theme(axis.line = element_blank(),
    axis.text.x  = element_blank(),
        axis.ticks.x= element_blank(),
        axis.title.x = element_text(size=20,face="bold"),
        axis.text.y =  element_text(size=18),
        axis.title.y= element_text(size=18,face="bold"),
        strip.text.x = element_text(size=18)) #+
p
```

We also fit a linear mixed-effects model estimating the average distance between item pairs for children from age (in years, as a continuous predictor; mean-centered), the category match for an image pair (same ESG category pair vs. different ESG category pair; centered), and their interaction. We included a by-participant random intercept and a by-participant random slope for ESG category match.

```
#### Summarizing Distances ####
#average distances across category types  within each participant
avg_dist_esg_by_subj <- subj_dist_long %>%
  group_by(subject, Age, Gender, age_bin,age_group,sort,esg_pair) %>%
  summarize(N=n(),avg_dist=mean(dist),ci_dist=qt(0.975, N-1)*sd(dist,na.rm=T)/sqrt(N))

avg_dist_esg_by_subj <- avg_dist_esg_by_subj %>%
  ungroup() %>%
  mutate(AgeC=Age-mean(Age,na.rm=T),
         esg_pairC=ifelse(esg_pair=="diff",-0.5,0.5),
         age_groupC=ifelse(age_group=="kids",-0.5,0.5),
         sortC=ifelse(sort=="Sort1",-0.5,0.5))

m <- lmer(avg_dist~esg_pairC*AgeC+(1+esg_pairC|subject),data=filter(avg_dist_esg_by_subj,sort!="Practice"&age_group=="kids"))
summary(m)
```

```
Linear mixed model fit by REML. t-tests use Satterthwaite's method [
lmerModLmerTest]
Formula: avg_dist ~ esg_pairC * AgeC + (1 + esg_pairC | subject)
   Data: filter(avg_dist_esg_by_subj, sort != "Practice" & age_group ==  
    "kids")

REML criterion at convergence: -1275.6

Scaled residuals: 
     Min       1Q   Median       3Q      Max 
-2.44626 -0.57749 -0.04332  0.64169  2.23074 

Random effects:
 Groups   Name        Variance  Std.Dev. Corr
 subject  (Intercept) 0.0003287 0.01813      
          esg_pairC   0.0016123 0.04015  0.33
 Residual             0.0020155 0.04489      
Number of obs: 422, groups:  subject, 106

Fixed effects:
                 Estimate Std. Error         df t value Pr(>|t|)    
(Intercept)      0.428634   0.002808 104.322117 152.674  < 2e-16 ***
esg_pairC       -0.053350   0.005860 103.971652  -9.104 6.74e-15 ***
AgeC            -0.002108   0.002549 104.127327  -0.827     0.41    
esg_pairC:AgeC  -0.034893   0.005320 103.781632  -6.558 2.17e-09 ***
---
Signif. codes:  0 '***' 0.001 '**' 0.01 '*' 0.05 '.' 0.1 ' ' 1

Correlation of Fixed Effects:
            (Intr) esg_pC AgeC 
esg_pairC   0.139              
AgeC        0.000  0.000       
esg_prC:AgC 0.000  0.000  0.139
```

The distances between images belonging to the same vs. different ESG categories increased with age (b = -0.03, Wald 95% CI = [-0.05, -0.02], F(1,103.78 = 43.01), p < .001). As children grow older, they become more likely to sort images belonging to the same emotion category closer together.

```
#3-year-olds
m_3 <- lmer(avg_dist~esg_pairC+(1+esg_pairC|subject),data=filter(avg_dist_esg_by_subj,age_bin=="3 to 4"&sort!="Practice"))
#4-year-olds
m_4  <- lmer(avg_dist~esg_pairC+(1+esg_pairC|subject),data=filter(avg_dist_esg_by_subj,age_bin=="4 to 5"&sort!="Practice"))
#5-year-olds
m_5 <- lmer(avg_dist~esg_pairC+(1+esg_pairC|subject),data=filter(avg_dist_esg_by_subj,age_bin=="5 to 6"&sort!="Practice"))
#6-year-olds
m_6 <- lmer(avg_dist~esg_pairC+(1+esg_pairC|subject),data=filter(avg_dist_esg_by_subj,age_bin=="6 to 7"&sort!="Practice"))
#adults
m_a <- lmer(avg_dist~esg_pairC+(1+esg_pairC|subject),data=filter(avg_dist_esg_by_subj,age_bin=="adults"&sort!="Practice"))
```

In follow-up analyses investigating children’s sorting behavior in each of our age groups, we found that 3-year-olds (p = 0.51) did not reliability sort images from the same ESG category closer together, while 4-year-olds (b = -0.02, Wald 95% CI = [-0.04, -0.01], F(1,99.08 = 10.56), p =0.002), 5-year-olds (b = -0.08, Wald 95% CI = [-0.11, -0.05], F(1,27 = 23.34), p < .001) and 6-year-olds did (b = -0.11, Wald 95% CI = [-0.14, -0.08], F(1,22 = 56.26), p < .001).

## Mouth Position

Each emotion category had one image with an open mouth and one image with a closed mouth. Since features of the mouth can sometimes influence emotion categorization in children (Caron, Caron & Myers, 1985) we investigated whether mouth position influenced sorting behaviors. Overall we did not find strong evidence that participants were using mouth position to guide their sorting behaviors.

```
# Plot
p <- ggplot(filter(avg_dist_diff_cat_across_subj, sort!="Practice"),aes(x=sort_name,y=average_dist,fill=age_bin,color=age_bin, linetype=sort_name))+
  geom_errorbar(aes(ymin=average_dist-ci_dist,ymax=average_dist+ci_dist),width=0,size=1)+
  geom_point(size=2.5)+
  facet_wrap(~age_bin,nrow=1,labeller = age_labeller, strip.position = c("bottom"))+
  geom_hline(yintercept=0)+
  theme(legend.position=c(.1,.8), legend.title = element_blank(),
        legend.margin = margin(.5,.5,.5,.5,"cm"),
        legend.background = element_rect(fill="white",size=0.6,
                                         linetype="solid", colour ="black")) +
  scale_color_manual(values=c("#8c510a","#bf812d","#80cdc1","#35978f","#01665e"))+
  guides(color=FALSE) +
  guides(fill=FALSE) +
  ylab("Sorting by Mouth Position\nLow<------------------------>High")+
  xlab("Age Group")+
  ylim(-.1,.3) +
  labs(linetype = "Sorting Phase") +
  theme(axis.line = element_blank(),
    axis.text.x  = element_blank(),
        axis.ticks.x= element_blank(),
        axis.title.x = element_text(size=20,face="bold"),
        axis.text.y =  element_text(size=18),
        axis.title.y= element_text(size=18,face="bold"),
        strip.text.x = element_text(size=18)) #+
p
```

## Actor Gender

For actor gender, we only examine the Different Individuals Sort (the Same Individual Sort was all one individual). Here, we look at whether an image was more likely to be placed by another image of the same gender. For instance, participants may have been more likely to place a female face with other female faces.

Overall we don’t find that participants are using actor gender to guide their behaviors. Adults were actually more likely to put actors of the same gender further apart from one another. We believe this is because adults were more likely to use emotion category to guide behavior. During the Different Individuals Sort, each emotion category had one female exemplar and one male exemplar. Thus, using actor gender to guide sorting behavior would cause individuals to use emotion category less.

```
p <- ggplot(filter(avg_dist_diff_cat_across_subj, sort!="Practice"),aes(x=sort_name,y=average_dist,fill=age_bin,color=age_bin, linetype=sort_name))+
  geom_errorbar(aes(ymin=average_dist-ci_dist,ymax=average_dist+ci_dist),width=0,size=1)+
  geom_point(size=1.5)+
  facet_wrap(~age_bin,nrow=1, labeller = age_labeller, strip.position = c("bottom"))+
  geom_hline(yintercept=0)+
  theme(legend.position=c(.1,.8), legend.title = element_blank(),
        legend.margin = margin(.5,.5,.5,.5,"cm"),
        legend.background = element_rect(fill="white",size=0.6,
                                         linetype="solid", colour ="black")) +
  scale_color_manual(values=c("#8c510a","#bf812d","#80cdc1","#35978f","#01665e"))+
  guides(color=FALSE) +
  guides(fill=FALSE) +
  ylab("Sorting by Actor Gender \nLow<------------------------>High")+
  xlab("Age Group")+
  ylim(-.1,.3) +
  theme(axis.line = element_blank(),
    axis.text.x  = element_blank(),
        axis.ticks.x= element_blank(),
        axis.title.x = element_text(size=20,face="bold"),
        axis.text.y =  element_text(size=18),
        axis.title.y= element_text(size=18,face="bold"),
        strip.text.x = element_text(size=18)) #+
p
```

## Resample w/ equal between and within observations

One possible concern with the above analyses is that there are an unequal numbers of distance observations for the same versus different category pairs: in particular, there are a far greater number of between-category than within-category distance observations. Does this bias the estimates of average distance between within- vs. between-category pairs systematically?

To investigate this question, we conducted an analysis in which we compared our original average distance estimates to estimates derived by resampling from the data such that only equal numbers of between and within observations were considered when computing average distance for within- vs. between-category pairs. For each resampled estimate, we randomly sampled (without replacement) 9 random between-category pairings (e.g., happy vs. sad) from each sort (Same Individual and Different Individual) and retained all 9 within-category pairings (e.g., happy vs. happy) from each sort. Thus, for each resampled dataset, there was an equal number of between-category and within-category observations among the 18 observations per participant and sort. For each age group and sorting condition, we then computed the difference between the average distance for between-category pairings and the average distance for within-category pairings to obtain a resampled estimate of the tendency to arrange images by category. We resampled from the dataset in this fashion 1000 times, thus obtaining 1000 resampled estimates of the category-based difference in sorting (see the analysis script 6\_resample\_category\_difference\_equal\_obs.R for the precise code).

The plot below compares these resampled estimates to the original estimate of the category-based sorting effect (based on the entire dataset), as reported in the main manuscript. We show each resampled estimate of the category-based sorting difference, along with violin plots to visualize their distribution. The large colored dots represent the mean of the resampled estimates and the original mean category-based sorting effect. We also show the 95% CI for the original category-based sorting estimate. The main takeaway from the plot is that the original estimate and the resampled estimates (always computed with an equal number of between-category and within-category estimates) are virtually identical. This indicates that the unequal numbers of between vs. within observations contributing to the original estimate are not systematically biasing the estimate of the category-based sorting effect.

```
#load the resampled data
summarized_resample_data <- read.csv( here(root_path,"analysis","paper_2020","processed_data","resampled_equal_obs_avg_category_distance.csv"))

#generate plot
# for facet wrap naming
age_names <- list(
  '3 to 4'="3 y/o",
  '4 to 5'="4 y/o",
  '5 to 6'="5 y/o",
  '6 to 7'="6 y/o",
  'adults'="adults")
age_labeller <- function(variable,value){
  return(age_names[value])
}

#recreate data from original emotion category plot
#focus on differences in dist (same v. between categories)
avg_dist_diff_cat_by_subj <- subj_dist_long %>%
  group_by(subject, Age, Gender, age_bin,age_group,sort,category_pair) %>%
  summarize(avg_dist=mean(dist,na.rm=T))  %>%
  ungroup() %>%
  group_by(subject, Age, Gender, age_bin,age_group,sort) %>%
  pivot_wider(names_from=category_pair,values_from=avg_dist) %>%
  mutate(avg_dist_diff=between-within) %>%
  mutate(sort_name=ifelse(sort=="Sort1","Same Individual",ifelse(sort=="Sort2","Different Individuals","Practice"))) %>%
  ungroup()

#average across subjects  by age bin
avg_dist_diff_cat_across_subj <- avg_dist_diff_cat_by_subj %>%
  select(-between,-within) %>%
  group_by(age_bin,age_group,sort,sort_name) %>%
  summarize(N=n(),average_dist=mean(avg_dist_diff,na.rm=T),ci_dist=qt(0.975, N-1)*sd(avg_dist_diff,na.rm=T)/sqrt(N))

# combine with the resampled data
comparison_data <- summarized_resample_data %>%
  mutate(resample_group="resampled") %>%
  bind_rows(filter(avg_dist_diff_cat_across_subj,sort!="Practice") %>% mutate(resample_group="original"))

p1 <- ggplot(filter(comparison_data, sort_name=="Same Individual"),aes(x=resample_group,y=average_dist,fill=age_bin,color=age_bin))+
  geom_violin(data=filter(comparison_data,resample_group=="resampled"&sort_name=="Same Individual"),fill=NA)+
  geom_jitter(data=filter(comparison_data,resample_group=="resampled"&sort_name=="Same Individual"),alpha=0.01,width=0.1)+
  geom_errorbar(data=filter(comparison_data,resample_group=="original"&sort_name=="Same Individual"),aes(ymin=average_dist-ci_dist,ymax=average_dist+ci_dist),width=0,size=1)+
  geom_point(data=filter(comparison_data,resample_group=="original"&sort_name=="Same Individual"),size=4,alpha=1)+
  stat_summary(data=filter(comparison_data,resample_group=="resampled"&sort_name=="Same Individual"),fun = "mean", geom = "point",size=4)+
  facet_wrap(~age_bin,nrow=1, labeller = age_labeller, strip.position = c("top"))+
  geom_hline(yintercept=0)+
  theme(legend.position=c(.1,.8), legend.title = element_blank(),
        legend.margin = margin(.5,.5,.5,.5,"cm"),
        legend.background = element_rect(fill="white",size=0.6,
                                         linetype="solid", colour ="black")) +
  #theme(legend.position='top', legend.justification ='center')+
  scale_color_manual(values=c("#8c510a","#bf812d","#80cdc1","#35978f","#01665e"))+
  scale_fill_manual(values=c("#8c510a","#bf812d","#80cdc1","#35978f","#01665e"))+
  guides(color=FALSE) +
  guides(fill=FALSE) +
  #scale_y_continuous(breaks=c(0,0.1,0.2,0.3, 0.4, 0.5,0.6), limits=c(0,0.5))+
  ylab("Sorting by Emotion Categories \nLow<----------------------------------->High")+
  xlab("Estimates")+
  labs(linetype = "Sorting Phase") +
  ggtitle("Same Individual Sort")+
  theme(#axis.line = element_blank(),
    #axis.text.x  = element_blank(),
    #axis.ticks.x= element_blank(),
    axis.text.x  = element_text(angle=90, vjust=0.5, size=10),
    axis.title.x = element_text(size=14,face="bold"),
    axis.text.y =  element_text(size=12),
    axis.title.y= element_text(size=12,face="bold"),
    strip.text.x = element_text(size=12),
    plot.title = element_text(hjust = 0.5,size=14))
p2 <- ggplot(filter(comparison_data, sort_name=="Different Individuals"),aes(x=resample_group,y=average_dist,fill=age_bin,color=age_bin))+
  geom_violin(data=filter(comparison_data,resample_group=="resampled"&sort_name=="Different Individuals"),fill=NA)+
  geom_jitter(data=filter(comparison_data,resample_group=="resampled"&sort_name=="Different Individuals"),alpha=0.01,width=0.1)+
  geom_errorbar(data=filter(comparison_data,resample_group=="original"&sort_name=="Different Individuals"),aes(ymin=average_dist-ci_dist,ymax=average_dist+ci_dist),width=0,size=1)+
  geom_point(data=filter(comparison_data,resample_group=="original"&sort_name=="Different Individuals"),size=4,alpha=1)+
  stat_summary(data=filter(comparison_data,resample_group=="resampled"&sort_name=="Different Individuals"),fun = "mean", geom = "point",size=4)+
  facet_wrap(~age_bin,nrow=1, labeller = age_labeller, strip.position = c("top"))+
  geom_hline(yintercept=0)+
  theme(legend.position=c(.1,.8), legend.title = element_blank(),
        legend.margin = margin(.5,.5,.5,.5,"cm"),
        legend.background = element_rect(fill="white",size=0.6,
                                         linetype="solid", colour ="black")) +
  #theme(legend.position='top', legend.justification ='center')+
  scale_color_manual(values=c("#8c510a","#bf812d","#80cdc1","#35978f","#01665e"))+
  scale_fill_manual(values=c("#8c510a","#bf812d","#80cdc1","#35978f","#01665e"))+
  guides(color=FALSE) +
  guides(fill=FALSE) +
  #scale_y_continuous(breaks=c(0,0.1,0.2,0.3, 0.4, 0.5,0.6), limits=c(0,0.5))+
  ylab("Sorting by Emotion Categories \nLow<----------------------------------->High")+
  xlab("Estimates")+
  labs(linetype = "Sorting Phase") +
  ggtitle("Different Individuals Sort")+
  theme(#axis.line = element_blank(),
    #axis.text.x  = element_blank(),
    #axis.ticks.x= element_blank(),
    axis.text.x  = element_text(angle=90, vjust=0.5, size=10),
    axis.title.x = element_text(size=14,face="bold"),
    axis.text.y =  element_text(size=12),
    axis.title.y= element_text(size=12,face="bold"),
    strip.text.x = element_text(size=12),
    plot.title = element_text(hjust = 0.5,size=14))
plot_grid(p1,p2,nrow=1,labels=c("A","B"),label_size=14)
```

## Correlations in category-based sorting between conditions

We also investigated whether the average distances at which pairs are placed are correlated across the 2 sets (Same vs. Different Individual). First, we correlated the distances between category-based item pairs (happy versus sad) across the two sorts. Second, we investigated category-based correlations at the participant level using a repeated measures correlation.

Across both analyses, we find small correlations for 3- and 4-year-olds. Category-based correlations between sorting conditions become increasingly robust among 5-year-olds and 6-year-olds, with the highest correlations among adults. This pattern mirrors the category-based sorting distance analyses, with 3- and 4-year-olds showing weak or no evidence for category-based sorting and increasing evidence for robust category-based sorting from the age of 5 onward.

### correlation between sorts

```
#pmap sorting command ensures that same category names are handled alphabetically across sorts. Ensures that ang-calm and calm-ang are handled as equivalent
subj_dist_by_pair <- subj_dist_long %>% 
  filter(sort!="Practice") %>%
  select(subject,age_bin,sort,dist,items,item1,item2,image_cat_1,image_cat_2,mouth1,mouth2) %>% 
  mutate(img_pairs = pmap_chr(list(image_cat_1,image_cat_2), ~paste(sort(c(...)), collapse = "_")))
#compute average distance by category pairing, age group, and sort
corr_mean_overall <- subj_dist_by_pair  %>% group_by(img_pairs,age_bin,sort) %>% summarise(distance=mean(dist,na.rm=TRUE))

#convert to wide format for easier computation of the correlation
corr_wide <- spread(corr_mean_overall,sort,distance)

#compute correlations for each age group
#then unlist into a wide data format for easier reporting
corr_age_group <- corr_wide %>%
  group_by(age_bin) %>%
  nest() %>%
  mutate(correlation_test=lapply(data, function(data) cor.test(data$Sort1,data$Sort2))) %>%
  mutate_if(is.list, simplify_all) %>%
  unnest_wider(correlation_test) %>%
  mutate(across(c("estimate.cor","conf.int1", "conf.int2", "statistic.t", "parameter.df", "p.value"),as.numeric)) %>%
  mutate(age =case_when(
    age_bin=="3 to 4" ~ "3-year-olds",
    age_bin=="4 to 5" ~ "4-year-olds",
    age_bin=="5 to 6" ~ "5-year-olds",
    age_bin=="6 to 7" ~ "6-year-olds",
    TRUE ~ "adults")) %>% 
  as.tibble()

#display table of outcomes
tab_corr<- corr_age_group %>%
  select(age, estimate.cor, conf.int1, conf.int2, statistic.t, parameter.df, p.value) %>%
  mutate(across(c("estimate.cor","conf.int1", "conf.int2", "statistic.t", "parameter.df"), round, 2)) %>%
  mutate(p.value=case_when(
    p.value>=.001 ~ as.character(round(p.value,3)),
    TRUE ~ "<.001"
  )) %>%
  mutate(conf=paste("[",conf.int1,", ",conf.int2,"]",sep="")) %>%
  relocate(conf, .after=estimate.cor) %>%
  select(-conf.int1, -conf.int2) %>%
  gt() %>% 
  cols_align(align="center") %>% 
  cols_label(age = md("**Age Groups**"), estimate.cor=md("**Correlation**"), conf=md("**95% CI**"),statistic.t=md("**t-value**"), parameter.df=md("**df**"), p.value=md("**p-value**")) %>%   
  tab_header(
    title = md("Correlations for category-based sorting distances between sorting conditions"))
tab_corr
```

| Correlations for category-based sorting distances between sorting conditions | | | | | |
| --- | --- | --- | --- | --- | --- |
| **Age Groups** | **Correlation** | **95% CI** | **t-value** | **df** | **p-value** |
| --- | --- | --- | --- | --- | --- |
| 3-year-olds | 0.39 | [0.11, 0.61] | 2.75 | 43 | 0.009 |
| 4-year-olds | 0.34 | [0.05, 0.57] | 2.34 | 43 | 0.024 |
| 5-year-olds | 0.60 | [0.37, 0.76] | 4.88 | 43 | <.001 |
| 6-year-olds | 0.67 | [0.47, 0.81] | 5.92 | 43 | <.001 |
| adults | 0.67 | [0.46, 0.8] | 5.87 | 43 | <.001 |

```
# plot
ggplot(corr_wide,aes(Sort1,Sort2,color=age_bin))+
  geom_point()+
  geom_smooth(method="lm")+
  scale_color_manual(values=c("#8c510a","#bf812d","#80cdc1","#35978f","#01665e"))+
  facet_wrap(~age_bin,nrow=1, labeller = age_labeller, strip.position = c("top"))+
  theme(legend.position="none")+
  xlab("Same Individual Sort")+
  ylab("Different Individuals Sort")
```

### repeated measures correlation between sorts

```
corr_subj_mean <- subj_dist_by_pair %>% 
  group_by(subject,age_bin,img_pairs,sort) %>% summarise(distance=mean(dist))

corr_subj_wide <- spread(corr_subj_mean,sort,distance) %>% 
  mutate(subject=as.factor(subject))

#compute correlations for each subject
#then unlist into a wide data format for easier reporting
corr_subj_age_group <- corr_subj_wide %>%
  group_by(age_bin) %>%
  nest() %>%
  mutate(rm_corr_plot=lapply(data, function(data) rmcorr(subject, Sort1, Sort2,dataset=data,CIs="analytic")),
    rm_corr=lapply(data, function(data) rmcorr(subject, Sort1, Sort2,dataset=data,CIs="analytic")[c("r","df","p","CI")])) %>%
  mutate_if(is.list, simplify_all) %>%
  unnest_wider(rm_corr) %>%
  mutate(age =case_when(
    age_bin=="3 to 4" ~ "3-year-olds",
    age_bin=="4 to 5" ~ "4-year-olds",
    age_bin=="5 to 6" ~ "5-year-olds",
    age_bin=="6 to 7" ~ "6-year-olds",
    TRUE ~ "adults")) %>% 
  as.tibble()

#display table of outcomes
tab_rmcorr<- corr_subj_age_group %>%
  select(age, r, CI1, CI2, df, p) %>%
  mutate(across(c("r","CI1", "CI2","df"), round, 2)) %>%
  mutate(p=case_when(
    p>=.001 ~ as.character(round(p,3)),
    TRUE ~ "<.001"
  )) %>%
  mutate(conf=paste("[",CI1,", ",CI2,"]",sep="")) %>%
  relocate(conf, .after=r) %>%
  select(-CI1, -CI2) %>%
  arrange(age) %>%
  gt() %>% 
  cols_align(align="center") %>% 
  cols_label(age = md("**Age Groups**"), r=md("**Correlation**"), conf=md("**95% CI**"), df=md("**df**"), p=md("**p-value**")) %>%   
  tab_header(
    title = md("Repeated Measures Correlations for category-based sorting distances between sorting conditions"))
tab_rmcorr
```

| Repeated Measures Correlations for category-based sorting distances between sorting conditions | | | | |
| --- | --- | --- | --- | --- |
| **Age Groups** | **Correlation** | **95% CI** | **df** | **p-value** |
| --- | --- | --- | --- | --- |
| 3-year-olds | 0.08 | [0.01, 0.14] | 923 | 0.016 |
| 4-year-olds | 0.03 | [-0.02, 0.08] | 1451 | 0.3 |
| 5-year-olds | 0.12 | [0.07, 0.18] | 1231 | <.001 |
| 6-year-olds | 0.22 | [0.16, 0.28] | 1011 | <.001 |
| adults | 0.36 | [0.32, 0.4] | 1759 | <.001 |

Plots of the repeated measures correlations within each age group can be viewed in the tabs below.

#### 3-year-olds

```
plot(filter(corr_subj_age_group,age=="3-year-olds")$rm_corr_plot[[1]], overall = TRUE,xlab="Same Individual Sort",ylab="Different Individuals Sort")
```

#### 4-year-olds

```
plot(filter(corr_subj_age_group,age=="4-year-olds")$rm_corr_plot[[1]], overall = TRUE,xlab="Same Individual Sort",ylab="Different Individuals Sort")
```

#### 5-year-olds

```
plot(filter(corr_subj_age_group,age=="5-year-olds")$rm_corr_plot[[1]], overall = TRUE,xlab="Same Individual Sort",ylab="Different Individuals Sort")
```

#### 6-year-olds

```
plot(filter(corr_subj_age_group,age=="6-year-olds")$rm_corr_plot[[1]], overall = TRUE,xlab="Same Individual Sort",ylab="Different Individuals Sort")
```

#### adults

```
plot(filter(corr_subj_age_group,age=="adults")$rm_corr_plot[[1]], overall = TRUE,xlab="Same Individual Sort",ylab="Different Individuals Sort")
```

# 3. Dimensions of Affect

```
dimension_data <- subj_dist_long %>% left_join(ratings_pairs)

dimension_data <- dimension_data %>% filter(sort!="Practice") %>% 
  select(subject, sort, Age, age_bin, age_group, dist, items, dist_valence, dist_arousal, dist_pos, dist_neg) %>%
  mutate(subject= as.factor(subject), age_group = as.factor(age_group), age_bin = as.factor(age_bin), sort=as.factor(sort)) %>% 
  mutate(sort2=case_when(
    sort == "Sort1" ~ "Same Individual",
    sort == "Sort2" ~ "Diff. Individual")) %>% 
  mutate(age_bin2=case_when(
    age_bin == '3 to 4' ~ "3 y/o",
    age_bin == '4 to 5' ~ "4 y/o",
    age_bin == '5 to 6' ~ "5 y/o",
    age_bin == '6 to 7' ~ "6 y/o",
    age_bin == 'adults' ~ "adults"))
```

# 3a. Adults versus Children

Here we display the relationship between the distance between two images based on their image ratings and the average distance that participants placed this images apart. If participants are using the rating of interest, we would expect that images with similar ratings are placed closer together and images with different ratings are placed further apart.

## Valence

```
ggplot(aes(x=dist_valence,y=dist,color=age_group,fill=age_group,linetype=sort2), data=dimension_data) + 
  geom_smooth(method="glm") +
  ylab("Average Distance Between Images")+
  xlab("Diff. in Valence Ratings Between Images")+
  labs(color="Age Group", linetype = "Sort") +
  guides(fill="none") +
  theme(axis.title.x = element_text(size=14,face="bold"),
        axis.text.y =  element_text(size=14),
        axis.title.y= element_text(size=14,face="bold"),
        strip.text.x = element_text(size=14))
```

## Arousal

```
ggplot(aes(x=dist_arousal,y=dist,color=age_group,fill=age_group,linetype=sort2), data=dimension_data) + 
  geom_smooth(method="glm") +
  ylab("Average Distance Between Images")+
  xlab("Diff. in Arousal Ratings Between Images")+
  labs(color="Age Group", linetype = "Sort") +
  guides(fill="none") +
  theme(axis.title.x = element_text(size=14,face="bold"),
        axis.text.y =  element_text(size=14),
        axis.title.y= element_text(size=14,face="bold"),
        strip.text.x = element_text(size=14))
```

## Positivity

```
ggplot(aes(x=dist_pos,y=dist,color=age_group,fill=age_group,linetype=sort2), data=dimension_data) + 
  geom_smooth(method="glm") +
  ylab("Average Distance Between Images")+
  xlab("Diff. in Positivity Ratings Between Images")+
  labs(color="Age Group", linetype = "Sort") +
  guides(fill="none") +
  theme(axis.title.x = element_text(size=14,face="bold"),
        axis.text.y =  element_text(size=14),
        axis.title.y= element_text(size=14,face="bold"),
        strip.text.x = element_text(size=14))
```

## Negativity

```
ggplot(aes(x=dist_neg,y=dist,color=age_group,fill=age_group,linetype=sort2), data=dimension_data) + 
  geom_smooth(method="glm") +
  ylab("Average Distance Between Images")+
  xlab("Diff. in Negativity Ratings Between Images")+
  labs(color="Age Group", linetype = "Sort") +
  guides(fill="none") +
  theme(axis.title.x = element_text(size=14,face="bold"),
        axis.text.y =  element_text(size=14),
        axis.title.y= element_text(size=14,face="bold"),
        strip.text.x = element_text(size=14))
```

# 3b. Children by Age Bin

Here we display the relationship between the distance between two images based on their image ratings and the average distance that participants placed this images apart. If participants are using the rating of interest, we would expect that images with similar ratings are placed closer together and images with different ratings are placed further apart.

## Valence

```
ggplot(aes(x=dist_valence,y=dist,color=age_bin2,fill=age_bin2), data=filter(dimension_data,age_bin!="adults")) + 
  geom_smooth(method="glm") +
  ylab("Average Distance Between Images")+
  xlab("Diff. in Valence Ratings Between Images")+
  labs(color="Age Group") +
  guides(fill="none") +
  theme(axis.title.x = element_text(size=14,face="bold"),
        axis.text.y =  element_text(size=14),
        axis.title.y= element_text(size=14,face="bold"),
        strip.text.x = element_text(size=14))
```

## Arousal

```
ggplot(aes(x=dist_arousal,y=dist,color=age_bin2,fill=age_bin2), data=filter(dimension_data,age_bin!="adults")) + 
  geom_smooth(method="glm") +
  ylab("Average Distance Between Images")+
  xlab("Diff. in Arousal Ratings Between Images")+
  labs(color="Age Group") +
  guides(fill="none") +
  theme(axis.title.x = element_text(size=14,face="bold"),
        axis.text.y =  element_text(size=14),
        axis.title.y= element_text(size=14,face="bold"),
        strip.text.x = element_text(size=14))
```

## Positivity

```
ggplot(aes(x=dist_pos,y=dist,color=age_bin2,fill=age_bin2), data=filter(dimension_data,age_bin!="adults")) + 
  geom_smooth(method="glm") +
  ylab("Average Distance Between Images")+
  xlab("Diff. in Positivity Ratings Between Images")+
  labs(color="Age Group") +
  guides(fill="none") +
  theme(axis.title.x = element_text(size=14,face="bold"),
        axis.text.y =  element_text(size=14),
        axis.title.y= element_text(size=14,face="bold"),
        strip.text.x = element_text(size=14))
```

## Negativity

```
ggplot(aes(x=dist_neg,y=dist,color=age_bin2,fill=age_bin2), data=filter(dimension_data,age_bin!="adults")) + 
  geom_smooth(method="glm") +
  ylab("Average Distance Between Images")+
  xlab("Diff. in Negativity Ratings Between Images")+
  labs(color="Age Group") +
  guides(fill="none") +
  theme(axis.title.x = element_text(size=14,face="bold"),
        axis.text.y =  element_text(size=14),
        axis.title.y= element_text(size=14,face="bold"),
        strip.text.x = element_text(size=14))
```

# 4. Model Comparisons

```
#load in data
drsq_v_a_ec <- read.csv(here(root_path,"analysis","paper_2020","processed_data","drsqr_v_a_ec.csv"))
drsq_pn_a_ec <- read.csv(here(root_path,"analysis","paper_2020","processed_data","drsqr_pn_a_ec.csv"))

drsq_v_a_ec <- drsq_v_a_ec %>% mutate(age_bin=case_when(
  age_bin == "3 to 4" ~ "3 y/o",
  age_bin == "4 to 5" ~ "4 y/o",
  age_bin == "5 to 6" ~ "5 y/o",
  age_bin == "6 to 7" ~ "6 y/o",
  age_bin == "adults" ~ "adults",
))

drsq_pn_a_ec <- drsq_pn_a_ec %>% mutate(age_bin=case_when(
  age_bin == "3 to 4" ~ "3 y/o",
  age_bin == "4 to 5" ~ "4 y/o",
  age_bin == "5 to 6" ~ "5 y/o",
  age_bin == "6 to 7" ~ "6 y/o",
  age_bin == "adults" ~ "adults",
))
```

## Valence plots

The left plot displays the full Model R-squared for each age group. The right plot displays the Delta R-squared for each predictor of sorting behavior. Error bars represent bootstrapped 95% confidence intervals.

```
pA <- ggplot(drsq_v_a_ec,aes(minus_model_name,delta_rsq,  fill=minus_model_name))+
  geom_bar(stat="identity",color="black")+
  geom_hline(yintercept=0,color="black")+
  geom_errorbar(aes(ymin=delta_rsq_lower,ymax=delta_rsq_upper),width=0.1)+
  scale_fill_viridis_d(
                    name = " ", #Model Predictor
                    limits=c("dist_valence","dist_arousal","emotion_pair_same"),
                    breaks=c("dist_valence","dist_arousal","emotion_pair_same"),
                    labels=c("Valence","Arousal","Emotion Category"))+
  scale_x_discrete(
    limits=c("dist_valence","dist_arousal","emotion_pair_same"),
    breaks=c("dist_valence","dist_arousal","emotion_pair_same"),
    labels=c("Valence","Arousal","Same Emotion Category"))+
  theme(axis.text.x  = element_text(angle=90, vjust=0.5,size=18),
        axis.title.x = element_text(size=14,face="bold"),
        axis.text.y =  element_text(size=14),
        axis.title.y= element_text(size=14,face="bold"),
        strip.text.x = element_text(size=14),
        legend.text =element_text(size=14),
        legend.title =element_text(size=16,face="bold"))+
  theme(axis.title.x=element_blank(),
        axis.text.x=element_blank(),
        axis.ticks.x=element_blank())+
  ylab("Delta R Squared")+
  facet_wrap(~age_bin,nrow=1, strip.position = "top")+
  theme(legend.position=c(0.1,0.8))

pB <- ggplot(unique(select(drsq_v_a_ec,age_bin,full_model_rsq)),aes(age_bin,full_model_rsq,  fill=age_bin))+
  geom_bar(stat="identity",color="black")+
  geom_hline(yintercept=0,color="black")+
  theme(axis.text.x  = element_text(angle=90, vjust=0.5,size=14),
        axis.title.x = element_text(size=14,face="bold"),
        axis.text.y =  element_text(size=14),
        axis.title.y= element_text(size=14,face="bold"),
        strip.text.x = element_text(size=14))+
  theme(axis.title.x=element_blank(),
        axis.text.x=element_blank(),
        axis.ticks.x=element_blank())+
  ylab("Model R Squared")+
  xlab("Age Group")+
theme(legend.position=c(0.1,0.8))
plot_grid(pB,pA)
```

## Pos-Neg plots

The left plot displays the full Model R-squared for each age group. The right plot displays the Delta R-squared for each predictor of sorting behavior. Error bars represent bootstrapped 95% confidence intervals.

```
pA <- ggplot(drsq_pn_a_ec,aes(minus_model_name,delta_rsq,  fill=minus_model_name))+
  geom_bar(stat="identity",color="black")+
  geom_hline(yintercept=0,color="black")+
  geom_errorbar(aes(ymin=delta_rsq_lower,ymax=delta_rsq_upper),width=0.1)+
  scale_fill_brewer(palette="Set1",
                    name = " ", #Model Predictor
                    limits=c("dist_pos","dist_neg","dist_arousal","emotion_pair_same"),
                    breaks=c("dist_pos","dist_neg","dist_arousal","emotion_pair_same"),
                    labels=c("Positive","Negative","Arousal","Same Emotion Category"))+
  scale_x_discrete(
    limits=c("dist_pos","dist_neg","dist_arousal","emotion_pair_same"),
    breaks=c("dist_pos","dist_neg","dist_arousal","emotion_pair_same"),
    labels=c("Positive","Negative","Arousal","Same Emotion Category"))+
  theme(axis.text.x  = element_text(angle=90, vjust=0.5,size=14),
        axis.title.x = element_text(size=14,face="bold"),
        axis.text.y =  element_text(size=14),
        axis.title.y= element_text(size=14,face="bold"),
        strip.text.x = element_text(size=14))+
  theme(axis.title.x=element_blank(),
        axis.text.x=element_blank(),
        axis.ticks.x=element_blank())+
  ylab("Delta R Squared")+
  facet_wrap(~age_bin,nrow=1)+
  theme(legend.position=c(0.1,0.9))

pB <- ggplot(unique(select(drsq_pn_a_ec,age_bin,full_model_rsq)),aes(age_bin,full_model_rsq,  fill=age_bin))+
  geom_bar(stat="identity",color="black")+
  geom_hline(yintercept=0,color="black")+
  theme(axis.text.x  = element_text(angle=90, vjust=0.5,size=18),
        axis.title.x = element_text(size=14,face="bold"),
        axis.text.y =  element_text(size=14),
        axis.title.y= element_text(size=14,face="bold"),
        strip.text.x = element_text(size=14))+
  theme(axis.title.x=element_blank(),
        axis.text.x=element_blank(),
        axis.ticks.x=element_blank())+
  ylab("Model R Squared")+
  xlab("Age Group")+
  theme(legend.position=c(0.1,0.8))
plot_grid(pB,pA)
```

# 5a. Multidimensional Scaling: Same Individual Sort

Here we present the same multidimensionsal scaling (MDS) results as in the manuscript. However, we provide additional visualizations. Reviewers can now see both faces and emotion category labels, as well as which cluster each face and image were a part of (from the hierarchical clustering k=3 solution)

```
cur_dist <- filter(avg_dist,sort=="Sort1"&age_group=="kids")$dist_obj[[1]]
sort1_kids_cmd <- data.frame(cmdscale(cur_dist, k=2))

# for image rating vectors
ratings1 <- ratings %>% filter(sort=="Sort1") %>%  select(image, pos, neg, valence, arousal) %>% rename(positivity=pos, negativity=neg)
sort1k_fit <- envfit(sort1_kids_cmd,ratings1, permutations = 1000)

# prepare images for plot
cur_cluster <- hclust(cur_dist)
image_paths <- paste(here(root_path,"experiment","stimuli_sort1"),"/",labels(cur_cluster),".png",sep="")
cur_images <- data.frame(
  label=labels(cur_cluster),
  image=image_paths,
  x=seq(1,length(image_paths)),
  y=rep(c(-0.05,-0.1),length(image_paths)/2))
sort1_images <- cur_images
cur_cmd <- data.frame(cmdscale(cur_dist))
cur_cmd$label <- rownames(cur_cmd)
cur_cmd <- merge(cur_cmd,sort1_images)

#include correlations at correct angles
data.scores = as.data.frame(scores(sort1_kids_cmd))
en_coord_cont = as.data.frame(scores(sort1k_fit, "vectors")) * ordiArrowMul(sort1k_fit)

#Plot MDS
p1 <- ggplot(data=cur_cmd,aes(X1,X2))+
  geom_image(data=cur_cmd, aes(image=image), size=0.08)+
  ylab("Dimension 2")+
  xlab("Dimension 1")+
  geom_segment(data = en_coord_cont,aes(x = 0, y = 0, xend = X1/3, yend = X2/3, color=row.names(en_coord_cont)), 
               size =1, alpha = 0.8, arrow = arrow(length = unit(0.03, "npc"))) + 
  geom_text(data = en_coord_cont, aes(x = X1/3, y = (X2/3) - .01), colour = "black",
            fontface = "bold", label = row.names(en_coord_cont))+
  theme(axis.text.x  = element_text(angle=90, vjust=0.5,size=18),
        axis.title.x = element_text(size=18,face="bold"),
        axis.text.y =  element_text(size=16),
        axis.title.y= element_text(size=18,face="bold"),
        strip.text.x = element_text(size=16))+
  ggtitle("Children")+
  theme(legend.position="none", plot.title = element_text(hjust = 0.5,size=18))

p1b <- ggplot(data=cur_cmd,aes(X1,X2))+
  geom_image(data=cur_cmd, aes(image=image), size=0.08)+
  ylab("Dimension 2")+
  xlab("Dimension 1")+
  annotate("rect", xmin=-.25,xmax=-.02, ymin=-.2,ymax=.2,alpha=.15,fill='red')+
  annotate("rect", xmin= .13,xmax=0.22, ymin=-.13,ymax=0,alpha=.15,fill='blue')+
  annotate("rect", xmin=-.02,xmax=0.13, ymin=-.05,ymax=.2,alpha=.15,fill='green')+
  theme(axis.text.x  = element_text(angle=90, vjust=0.5,size=18),
        axis.title.x = element_text(size=18,face="bold"),
        axis.text.y =  element_text(size=16),
        axis.title.y= element_text(size=18,face="bold"),
        strip.text.x = element_text(size=16))+
  ggtitle("Children")+
  theme(legend.position="none", plot.title = element_text(hjust = 0.5,size=18))

#adults
cur_dist <- filter(avg_dist,sort=="Sort1"&age_group=="adults")$dist_obj[[1]]
sort1_adults_cmd <- data.frame(cmdscale(cur_dist, k=2))

# for image rating vectors
sort1a_fit <- envfit(sort1_adults_cmd,ratings1, permutations=1000)
data.scores = as.data.frame(scores(sort1_adults_cmd))
en_coord_cont = as.data.frame(scores(sort1a_fit, "vectors")) * ordiArrowMul(sort1a_fit)

#prepare images
cur_cmd <- data.frame(cmdscale(cur_dist))
cur_cmd$label <- rownames(cur_cmd)
cur_cmd <- merge(cur_cmd,sort1_images)

#Plot MDS
p2 <- ggplot(data=cur_cmd,aes(X1,X2))+
  geom_image(data=cur_cmd, aes(image=image), size=0.08)+
  ylab("Dimension 2")+
  xlab("Dimension 1")+
  geom_segment(data = en_coord_cont,aes(x = 0, y = 0, xend = X1/3, yend = X2/3, color=row.names(en_coord_cont)), 
               size =1, alpha = 0.8, arrow = arrow(length = unit(0.03, "npc"))) + #ends="both" for arrow
  geom_text(data = en_coord_cont, aes(x = X1/3, y = (X2/3) - .01), colour = "black",
            fontface = "bold", label = row.names(en_coord_cont))+
  theme(axis.text.x  = element_text(angle=90, vjust=0.5,size=18),
        axis.title.x = element_text(size=18,face="bold"),
        axis.text.y =  element_text(size=16),
        axis.title.y= element_text(size=18,face="bold"),
        strip.text.x = element_text(size=16))+
  ggtitle("Adults")+
  theme(legend.position="none", plot.title = element_text(hjust = 0.5,size=18))

p2b <- ggplot(data=cur_cmd,aes(X1,X2))+
  geom_image(data=cur_cmd, aes(image=image), size=0.08)+
  ylab("Dimension 2")+
  xlab("Dimension 1")+
  annotate("rect", xmin=-.35,xmax=-.14, ymin=-.07,ymax=.2,alpha=.15,fill='red')+
  annotate("rect", xmin=.15,xmax=0.4, ymin=-.07,ymax=.2,alpha=.15,fill='blue')+
  annotate("rect", xmin=-.2,xmax=0.18, ymin=-.25,ymax=-.07,alpha=.15,fill='green')+
  theme(axis.text.x  = element_text(angle=90, vjust=0.5,size=18),
        axis.title.x = element_text(size=18,face="bold"),
        axis.text.y =  element_text(size=16),
        axis.title.y= element_text(size=18,face="bold"),
        strip.text.x = element_text(size=16))+
  ggtitle("Adults")+
  theme(legend.position="none", plot.title = element_text(hjust = 0.5,size=18))
```

```
library(ggrepel)

#Children
cur_dist <- filter(avg_dist,sort=="Sort1"&age_group=="kids")$dist_obj[[1]]
sort1_kids_cmd <- data.frame(cmdscale(cur_dist, k=2))
cur_cmd <- data.frame(cmdscale(cur_dist))
cur_cmd$label <- rownames(cur_cmd)
cur_cmd <- merge(cur_cmd,sort1_images)

s1k <- hclust(cur_dist, method = "ward.D2") #get hclust solution
s1k <- as.data.frame(cutree(s1k, 3))
s1k <- s1k %>% tibble::rownames_to_column("label") %>% rename(cluster3 = `cutree(s1k, 3)`)
s1k <- s1k %>% mutate(clean_label =
  str_replace_all(label, c("M07"="","_o"=" 1", "_c"=" 2", "ang"="angry","calm"="calm","disg"="disgust",
                    "exc"="excited","fear"="fear", "hap"="happy",
                    "neut"="neutral","sad"="sad", "surp"="surprise")))

cur_cmd <- cur_cmd %>%  #combine hclust and mds
  dplyr:::inner_join(s1k, by = c("label"))

p1c <- ggplot(data=cur_cmd,aes(X1,X2,label=clean_label,color=as.factor(cluster3)))+
  geom_text_repel(fontface = "bold", min.segment.length = .3)+
  ylab("Dimension 2")+
  xlab("Dimension 1")+
  theme(axis.text.x  = element_text(angle=90, vjust=0.5,size=18),
        axis.title.x = element_text(size=18,face="bold"),
        axis.text.y =  element_text(size=16),
        axis.title.y= element_text(size=18,face="bold"),
        strip.text.x = element_text(size=16))+
  ggtitle("Children")+
  theme(legend.position="none", plot.title = element_text(hjust = 0.5,size=18))

#Adults
cur_dist <- filter(avg_dist,sort=="Sort1"&age_group=="adults")$dist_obj[[1]]
sort1_adults_cmd <- data.frame(cmdscale(cur_dist, k=2))
cur_cmd <- data.frame(cmdscale(cur_dist))
cur_cmd$label <- rownames(cur_cmd)
cur_cmd <- merge(cur_cmd,sort1_images)

s1a <- hclust(cur_dist, method = "ward.D2") #get hclust solution
s1a <- as.data.frame(cutree(s1a, 3))
s1a <- s1a %>% tibble::rownames_to_column("label") %>% rename(cluster3 = `cutree(s1a, 3)`)
s1a <- s1a %>% mutate(clean_label =
  str_replace_all(label, c("M07"="","_o"=" 1", "_c"=" 2", "ang"="angry","calm"="calm","disg"="disgust",
                    "exc"="excited","fear"="fear", "hap"="happy",
                    "neut"="neutral","sad"="sad", "surp"="surprise")))

cur_cmd <- cur_cmd %>%  #combine hclust and mds
  dplyr:::inner_join(s1a, by = c("label"))

#changing for color reasons only
cur_cmd <- cur_cmd %>% mutate(cluster3=case_when(
  cluster3 == 1~1,
  cluster3 == 2~3,
  cluster3 == 3~2))

p2c <- ggplot(data=cur_cmd,aes(X1,X2,label=clean_label,color=as.factor(cluster3)))+
  geom_text_repel(fontface = "bold", min.segment.length = .3)+
  ylab("Dimension 2")+
  xlab("Dimension 1")+
  theme(axis.text.x  = element_text(angle=90, vjust=0.5,size=18),
        axis.title.x = element_text(size=18,face="bold"),
        axis.text.y =  element_text(size=16),
        axis.title.y= element_text(size=18,face="bold"),
        strip.text.x = element_text(size=16))+
  ggtitle("Adults")+
  theme(legend.position="none", plot.title = element_text(hjust = 0.5,size=18))
```

## Faces with image ratings

```
plot_grid(p1,p2,labels=c("A","B"),label_size=20)
```

## Faces with Hierarchical Clustering solution

```
plot_grid(p1b,p2b,labels=c("A","B"),label_size=20)
```

Colors highlight k=3 hierarchical clustering solutions for each age group. We used the three colors to highlight commonalities among the different hierarchical clustering solutions. For instance, red clusters contain anger and disgust images, green clusters contain certain fear and neutral images, and blue images contain certain happy, calm, and surprise images. However, there was nothing statistically to indicate “this is the red cluster” across all dendrograms.

## Labels with Hierarchical Clustering solution

```
plot_grid(p1c,p2c,labels=c("A","B"),label_size=20)
```

Colors highlight the three cluster solutions for each age-bin. We used the three colors to highlight commonalities among the different hierarchical clustering solutions. For instance, red clusters contain anger and disgust images, green clusters contain certain fear and neutral images, and blue images contain certain happy, calm, and surprise images. However, there was nothing statistically to indicate “this is the red cluster” across all dendrograms. The numbers 1 and 2 indicate that the images had open and closed mouths, respectively. Labels are slightly jittered for readability (indicated by lines).

# 5b. Multidimensional Scaling: Different Individuals Sort

Here we present the same multidimensionsal scaling (MDS) results as in the manuscript. However, we provide additional visualizations. Reviewers can now see both faces and emotion category labels, as well as which cluster each face and image were a part of (from the hierarchical clustering k=3 solution)

```
#### Children ####
cur_dist <- filter(avg_dist,sort=="Sort2"&age_group=="kids")$dist_obj[[1]]
sort2_kids_cmd <- data.frame(cmdscale(cur_dist, k=2))

# get vectors of image ratings
ratings2 <- ratings %>% filter(sort=="Sort2") %>%  select(image, pos, neg, valence, arousal) %>% rename(positivity=pos, negativity=neg)
sort2k_fit <- envfit(sort2_kids_cmd,ratings2, permutations=1000)
data.scores = as.data.frame(scores(sort2_kids_cmd))
en_coord_cont = as.data.frame(scores(sort2k_fit, "vectors")) * ordiArrowMul(sort2k_fit)

#create image paths
cur_cluster <- hclust(cur_dist)
image_paths <- paste(here(root_path,"experiment","stimuli_sort2"),"/",labels(cur_cluster),".png",sep="")
cur_images <- data.frame(
  label=labels(cur_cluster),
  image=image_paths,
  x=seq(1,length(image_paths)),
  y=rep(c(-0.05,-0.1),length(image_paths)/2))
sort2_images <- cur_images
cur_cmd <- data.frame(cmdscale(cur_dist))
cur_cmd$label <- rownames(cur_cmd)
cur_cmd <- merge(cur_cmd,sort2_images)

#Plot MDS
p1 <- ggplot(data=cur_cmd,aes(X1,X2))+
  geom_image(data=cur_cmd, aes(image=image), size=0.08)+
  ylab("Dimension 2")+
  xlab("Dimension 1")+
  geom_segment(data = en_coord_cont,aes(x = 0, y = 0, xend = X1/3, yend = X2/3, color=row.names(en_coord_cont)), 
               size =1, alpha = 0.8, arrow = arrow(length = unit(0.03, "npc"))) + #ends="both" for arrow
  geom_text(data = en_coord_cont, aes(x = X1/3, y = (X2/3) - .01), colour = "black",
            fontface = "bold", label = row.names(en_coord_cont))+
  theme(axis.text.x  = element_text(angle=90, vjust=0.5,size=18),
        axis.title.x = element_text(size=18,face="bold"),
        axis.text.y =  element_text(size=16),
        axis.title.y= element_text(size=18,face="bold"),
        strip.text.x = element_text(size=16))+
  ggtitle("Children")+
  theme(legend.position="none", plot.title = element_text(hjust = 0.5,size=18))

p1b <- ggplot(data=cur_cmd,aes(X1,X2))+
  geom_image(data=cur_cmd, aes(image=image), size=0.08)+
  ylab("Dimension 2")+
  xlab("Dimension 1")+
  annotate("rect", xmin=-.26,xmax=-.12, ymin=-.18,ymax=.12,alpha=.15,fill='red')+
  annotate("rect", xmin=0.05,xmax=0.2, ymin=-.2,ymax=0.05,alpha=.15,fill='blue')+
  annotate("rect", xmin=-.05,xmax=0.12, ymin=0.05,ymax=.2,alpha=.15,fill='green')+
  theme(axis.text.x  = element_text(angle=90, vjust=0.5,size=18),
        axis.title.x = element_text(size=18,face="bold"),
        axis.text.y =  element_text(size=16),
        axis.title.y= element_text(size=18,face="bold"),
        strip.text.x = element_text(size=16))+
  ggtitle("Children")+
  theme(legend.position="none", plot.title = element_text(hjust = 0.5,size=18))


#### Adults ####
cur_dist <- filter(avg_dist,sort=="Sort2"&age_group=="adults")$dist_obj[[1]]
sort2_adults_cmd <- data.frame(cmdscale(cur_dist, k=2))

# for image rating vectors
sort2a_fit <- envfit(sort2_adults_cmd,ratings2, permutations=1000)
data.scores = as.data.frame(scores(sort2_adults_cmd))
en_coord_cont = as.data.frame(scores(sort2a_fit, "vectors")) * ordiArrowMul(sort2a_fit)

# prepare images
cur_cmd <- data.frame(cmdscale(cur_dist))
cur_cmd$label <- rownames(cur_cmd)
cur_cmd <- merge(cur_cmd,sort2_images)

#Plot MDS
p2 <- ggplot(data=cur_cmd,aes(X1,X2))+
  geom_image(data=cur_cmd, aes(image=image), size=0.08)+
  ylab("Dimension 2")+
  xlab("Dimension 1")+
  geom_segment(data = en_coord_cont,aes(x = 0, y = 0, xend = X1/3, yend = X2/3, color=row.names(en_coord_cont)), 
               size =1, alpha = 0.8, arrow = arrow(length = unit(0.03, "npc"))) + #ends="both" for arrow
  geom_text(data = en_coord_cont, aes(x = X1/3, y = (X2/3) - .02), colour = "black",
            fontface = "bold", label = row.names(en_coord_cont))+
  theme(axis.text.x  = element_text(angle=90, vjust=0.5,size=18),
        axis.title.x = element_text(size=18,face="bold"),
        axis.text.y =  element_text(size=16),
        axis.title.y= element_text(size=18,face="bold"),
        strip.text.x = element_text(size=16))+
  ggtitle("Adults")+
  theme(legend.position="none", plot.title = element_text(hjust = 0.5,size=18))

p2b <- ggplot(data=cur_cmd,aes(X1,X2))+
  geom_image(data=cur_cmd, aes(image=image), size=0.08)+
  ylab("Dimension 2")+
  xlab("Dimension 1")+
  annotate("rect", xmin=-.4,xmax=-.27, ymin=-.13,ymax=0.02,alpha=.15,fill='blue')+
  annotate("rect", xmin=.19,xmax=0.35, ymin=-.25,ymax=-.12,alpha=.15,fill='red')+
  annotate("rect", xmin=-.17,xmax=0.20, ymin=-0.11,ymax=.31,alpha=.15,fill='green')+
  theme(axis.text.x  = element_text(angle=90, vjust=0.5,size=18),
        axis.title.x = element_text(size=18,face="bold"),
        axis.text.y =  element_text(size=16),
        axis.title.y= element_text(size=18,face="bold"),
        strip.text.x = element_text(size=16))+
  ggtitle("Adults")+
  theme(legend.position="none", plot.title = element_text(hjust = 0.5,size=18))
```

```
library(ggrepel)

#Children
cur_dist <- filter(avg_dist,sort=="Sort2"&age_group=="kids")$dist_obj[[1]]
sort2_kids_cmd <- data.frame(cmdscale(cur_dist, k=2))
cur_cmd <- data.frame(cmdscale(cur_dist))
cur_cmd$label <- rownames(cur_cmd)
cur_cmd <- merge(cur_cmd,sort2_images)

s2k <- hclust(cur_dist, method = "ward.D2") #get hclust solution
s2k <- as.data.frame(cutree(s2k, 3))
s2k <- s2k %>% tibble::rownames_to_column("label") %>% rename(cluster3 = `cutree(s2k, 3)`)
s2k <- s2k %>% mutate(clean_label =
  str_replace_all(label, c("F01"="","F04"="","F07"="","F10"="", "F13"="",
                    "F14"="","F15"="", "F17"="","F22"="","M02"="","M04"="",
                    "M03"="","M05"="","M08"="","M12"="","M14"="","M15"="",
                    "M17"="","_o"=" 1", "_c"=" 2", "ang"="angry", "calm"="calm",
                    "disg"="disgust", "exc"="excited", "fear"="fear",
                    "hap"="happy", "neut"="neutral", "sad"="sad",
                    "surp"="surprise")))

cur_cmd <- cur_cmd %>% #combine hclust and mds
  dplyr:::inner_join(s2k, by = c("label"))

#changing for color reasons only
cur_cmd <- cur_cmd %>% mutate(cluster3=case_when(
  cluster3 == 1~3,
  cluster3 == 2~1,
  cluster3 == 3~2))

p1c <- ggplot(data=cur_cmd,aes(X1,X2,label=clean_label,color=as.factor(cluster3)))+
  geom_text_repel(fontface = "bold", min.segment.length = .3)+
  ylab("Dimension 2")+
  xlab("Dimension 1")+
  theme(axis.text.x  = element_text(angle=90, vjust=0.5,size=18),
        axis.title.x = element_text(size=18,face="bold"),
        axis.text.y =  element_text(size=16),
        axis.title.y= element_text(size=18,face="bold"),
        strip.text.x = element_text(size=16))+
  ggtitle("Children")+
  theme(legend.position="none", plot.title = element_text(hjust = 0.5,size=18))

#Adults
cur_dist <- filter(avg_dist,sort=="Sort2"&age_group=="adults")$dist_obj[[1]]
sort2_adults_cmd <- data.frame(cmdscale(cur_dist, k=2))
cur_cmd <- data.frame(cmdscale(cur_dist))
cur_cmd$label <- rownames(cur_cmd)
cur_cmd <- merge(cur_cmd,sort2_images)

s2a <- hclust(cur_dist, method = "ward.D2") #get hclust solution
s2a <- as.data.frame(cutree(s2a, 3))
s2a <- s2a %>% tibble::rownames_to_column("label") %>% rename(cluster3 = `cutree(s2a, 3)`)
s2a <- s2a %>% mutate(clean_label =
  str_replace_all(label, c("F01"="","F04"="","F07"="","F10"="", "F13"="",
                    "F14"="","F15"="", "F17"="","F22"="","M02"="","M04"="",
                    "M03"="","M05"="","M08"="","M12"="","M14"="","M15"="",
                    "M17"="","_o"=" 1", "_c"=" 2", "ang"="angry", "calm"="calm",
                    "disg"="disgust", "exc"="excited", "fear"="fear",
                    "hap"="happy", "neut"="neutral", "sad"="sad",
                    "surp"="surprise")))

cur_cmd <- cur_cmd %>% #combine hclust and mds
  dplyr:::inner_join(s2a, by = c("label"))

#changing for color reasons only
cur_cmd <- cur_cmd %>% mutate(cluster3=case_when(
  cluster3 == 1~2,
  cluster3 == 2~1,
  cluster3 == 3~3))

p2c <- ggplot(data=cur_cmd,aes(X1,X2,label=clean_label,color=as.factor(cluster3)))+
  geom_text_repel(fontface = "bold", min.segment.length = .3)+
  ylab("Dimension 2")+
  xlab("Dimension 1")+
  theme(axis.text.x  = element_text(angle=90, vjust=0.5,size=18),
        axis.title.x = element_text(size=18,face="bold"),
        axis.text.y =  element_text(size=16),
        axis.title.y= element_text(size=18,face="bold"),
        strip.text.x = element_text(size=16))+
  ggtitle("Adults")+
  theme(legend.position="none", plot.title = element_text(hjust = 0.5,size=18))
```

## Faces with image ratings

```
plot_grid(p1,p2,labels=c("C","D"),label_size=20)
```

## Faces with Hierarchical Clustering solution

```
plot_grid(p1b,p2b,labels=c("C","D"),label_size=20)
```

Colors highlight k=3 hierarchical clustering solutions for each age group. We used the three colors to highlight commonalities among the different hierarchical clustering solutions. For instance, red clusters contain anger and disgust images, green clusters contain certain fear and neutral images, and blue images contain certain happy, calm, and surprise images. However, there was nothing statistically to indicate “this is the red cluster” across all dendrograms.

## Labels with Hierarchical Clustering solution

```
plot_grid(p1c,p2c,labels=c("A","B"),label_size=20)
```

Colors highlight the three cluster solutions for each age-bin. We used the three colors to highlight commonalities among the different hierarchical clustering solutions. For instance, red clusters contain anger and disgust images, green clusters contain certain fear and neutral images, and blue images contain certain happy, calm, and surprise images. However, there was nothing statistically to indicate “this is the red cluster” across all dendrograms. The numbers 1 and 2 indicate that the images had open and closed mouths, respectively. Labels are slightly jittered for readability (indicated by lines).

# 5c. Multidimensional Scaling: How many dimensions?

We found that the 2-dimensional MDS solution had the best fit. We also felt that the 2-dimensional structure was most appropriate given the nature of the task (e.g., sorting all images together in a 2-dimensional space)

```
m1 <- data.frame(mds1$eig)
m2 <- data.frame(mds2$eig)
m3 <- data.frame(mds3$eig)
m4 <- data.frame(mds4$eig)

m1 <- m1 %>% rownames_to_column() %>% rename(num_dim=rowname, eig=mds1.eig) %>% mutate(sort="Same Individual",age="Children")
m2 <- m2 %>% rownames_to_column() %>% rename(num_dim=rowname, eig=mds2.eig) %>% mutate(sort="Same Individual",age="Adults")
m3 <- m3 %>% rownames_to_column() %>% rename(num_dim=rowname, eig=mds3.eig) %>% mutate(sort="Different Individuals",age="Children")
m4 <- m4 %>% rownames_to_column() %>% rename(num_dim=rowname, eig=mds4.eig) %>% mutate(sort="Different Individuals",age="Adults")
m_full <- rbind(m1,m2,m3,m4)
m_full$num_dim=as.numeric(m_full$num_dim)
```

```
p <- ggplot(data=m_full,aes(x=num_dim,y=eig,fill=age, color=age)) + 
  geom_area() +
  facet_wrap(~sort) +
  geom_vline(xintercept=2)+
  ylab("Eigenvalue")+
  xlab("Number of Dimensions") +
  scale_x_continuous(breaks=c(1,2,3,4,5,6,7,8,9,11,13,15,17))
 p
```

# 6a. Hierarchical Clustering: Additional Dendrograms

We present additional dendrograms comparison adults and children (collapsing across all age groups). A dendrogram is a way of visualizing a hierarchical clustering solution. It displays all clustering solutions - in our case, from k =2 to k =18. Each face participants sorted is a “node” on the dendrogram and when it merges with another face (represented by a horizontal line) another “node” is formed. Clusters are determined by the vertical height of the branches in a dendrogram, not by which labels are closest to one another laterally. Thus, faces that were found to be the most similar would be connected as a node with a very low height. The k = 3 cluster that we display shows what happens if a horizontal line was drawn that only passed through 3 vertical lines.

Colors highlight the three cluster solutions for each age-bin. We used the three colors to highlight commonalities among the different dendrograms. For instance, red clusters contain anger and disgust images, green clusters contain certain fear and neutral images, and blue images contain certain happy, calm, and surprise images. However, there was nothing statistically to indicate “this is the red cluster” across all dendrograms. The numbers 1 and 2 indicate that the images had open and closed mouths, respectively.

```
#average across all distances
avg_dist_long_byGroup <- subj_dist_long %>%
  group_by(sort,age_group,item1,item2) %>%
  summarize(avg_dist=mean(dist)) %>%
  ungroup() %>%
  mutate(sort=as.character(sort),age_group=as.character(age_group),item1=as.character(item1),item2=as.character(item2))

#average distance object organized by sorting group
avg_dist_byGroup <- avg_dist_long_byGroup %>%
  group_by(sort,age_group) %>%
  nest() %>%
  mutate(dist_obj = map(data, long_to_dist))

#average across all distances
avg_dist_long_byBin <- subj_dist_long %>%
  group_by(sort,age_bin,item1,item2) %>%
  summarize(avg_dist=mean(dist)) %>%
  ungroup() %>%
  mutate(sort=as.character(sort),age_bin=as.character(age_bin),item1=as.character(item1),item2=as.character(item2))

#average distance object organized by sorting group
avg_dist_byBin <- avg_dist_long_byBin %>%
  group_by(sort,age_bin) %>%
  nest() %>%
  mutate(dist_obj = map(data, long_to_dist))
```

## Same Individual Sort: Adults and Children

```
#distance matrix objects
s1k_dist <- avg_dist_byGroup %>%
  dplyr::filter(sort=="Sort1"&age_group=="kids") %>%
  ungroup() %>%
  select(dist_obj)
s1k_dist <- s1k_dist$dist_obj[[1]]

s1a_dist <- avg_dist_byGroup %>%
  dplyr::filter(sort=="Sort1"&age_group=="adults") %>%
  ungroup() %>%
  select(dist_obj)
s1a_dist <- s1a_dist$dist_obj[[1]]

# hclust solution
s1k <- s1k_dist %>%
  hclust(method = "ward.D2")

s1a <- s1a_dist %>%
  hclust(method = "ward.D2")

#clean up labels
s1k[["labels"]] <- s1k[["labels"]] %>%
  str_replace_all(c("M07"="","_o"=" 1", "_c"=" 2", "ang"="angry","calm"="calm","disg"="disgust",
                    "exc"="excited","fear"="fear", "hap"="happy",
                    "neut"="neutral","sad"="sad", "surp"="surprise"))

s1a[["labels"]] <- s1a[["labels"]] %>%
  str_replace_all(c("M07"="","_o"=" 1", "_c"=" 2", "ang"="angry","calm"="calm","disg"="disgust",
                    "exc"="excited","fear"="fear", "hap"="happy",
                    "neut"="neutral","sad"="sad", "surp"="surprise"))

# dendrograms
s1k_dend <- s1k %>% as.dendrogram
s1a_dend <- s1a %>% as.dendrogram

# plots
par(mfrow = c(1,2))
s1a_dend %>% set("branches_lwd", 2) %>%  set("branches_k_color",value=c(4,3,2), k=3) %>% plot(main="Adults")
s1k_dend %>% set("branches_lwd", 2) %>%  set("branches_k_color",value=c(2,4,3), k=3)%>% plot(main="Children")
```

## Different Individuals Sort: Adults and Children

```
s2k_dist <- avg_dist_byGroup %>%
  dplyr::filter(sort=="Sort2"&age_group=="kids") %>%
  ungroup() %>%
  select(dist_obj)
s2k_dist <- s2k_dist$dist_obj[[1]]

s2a_dist <- avg_dist_byGroup %>%
  dplyr::filter(sort=="Sort2"&age_group=="adults") %>%
  ungroup() %>%
  select(dist_obj)
s2a_dist <- s2a_dist$dist_obj[[1]]

#hclust objects
s2k <- s2k_dist %>%
  hclust(method = "ward.D2")

s2a <- s2a_dist %>%
  hclust(method = "ward.D2")


#clean up labels
s2k[["labels"]] <- s2k[["labels"]] %>%
  str_replace_all(c("F01"="","F04"="","F07"="","F10"="", "F13"="",
                    "F14"="","F15"="", "F17"="","F22"="","M02"="","M04"="",
                    "M03"="","M05"="","M08"="","M12"="","M14"="","M15"="",
                    "M17"="","_o"=" 1", "_c"=" 2", "ang"="angry", "calm"="calm",
                    "disg"="disgust", "exc"="excited", "fear"="fear",
                    "hap"="happy", "neut"="neutral", "sad"="sad",
                    "surp"="surprise"))

s2a[["labels"]] <- s2a[["labels"]] %>%
  str_replace_all(c("F01"="","F04"="","F07"="","F10"="", "F13"="",
                    "F14"="","F15"="", "F17"="","F22"="","M02"="","M04"="",
                    "M03"="","M05"="","M08"="","M12"="","M14"="","M15"="",
                    "M17"="","_o"=" 1", "_c"=" 2", "ang"="angry", "calm"="calm",
                    "disg"="disgust", "exc"="excited", "fear"="fear",
                    "hap"="happy", "neut"="neutral", "sad"="sad",
                    "surp"="surprise"))

s2k_dend <- s2k %>% as.dendrogram
s2a_dend <- s2a %>% as.dendrogram

#Dendrograms
par(mfrow = c(1,2))
s2a_dend %>% set("branches_lwd", 2) %>%  set("branches_k_color",value=c(4,2,3), k=3) %>% plot(main="Adults")
s2k_dend %>% set("branches_lwd", 2) %>%  set("branches_k_color",value=c(2,4,3), k=3) %>% plot(main="Children")
```

# 6b. Similarity Indices

Here we provide additional indices of similarity between hierarchical cluster solutions and for all possible k values. While the manuscript primarily reported on the three cluster solution, we understand selecting the number of clusters is inexact. For this reason, we give additional similarity measures across all cluster sizes.

```
# Same Individual Sort
s1_3 <- avg_dist_byBin %>%
  dplyr::filter(sort=="Sort1"&age_bin=="3 to 4") %>%
  ungroup() %>%
  select(dist_obj)

s1_4 <- avg_dist_byBin %>%
  dplyr::filter(sort=="Sort1"&age_bin=="4 to 5") %>%
  ungroup() %>%
  select(dist_obj)

s1_5 <- avg_dist_byBin %>%
  dplyr::filter(sort=="Sort1"&age_bin=="5 to 6") %>%
  ungroup() %>%
  select(dist_obj)

s1_6 <- avg_dist_byBin %>%
  dplyr::filter(sort=="Sort1"&age_bin=="6 to 7") %>%
  ungroup() %>%
  select(dist_obj)

s1_a <- avg_dist_byBin %>%
  dplyr::filter(sort=="Sort1"&age_bin=="adults") %>%
  ungroup() %>%
  select(dist_obj)

#clean dist object
s1_3_dist <- s1_3$dist_obj[[1]]

s1_4_dist  <- s1_4$dist_obj[[1]]

s1_5_dist  <- s1_5$dist_obj[[1]]

s1_6_dist  <- s1_6$dist_obj[[1]] 

s1_a_dist  <- s1_a$dist_obj[[1]]

#hclust
s1_3 <- s1_3$dist_obj[[1]] %>%
  hclust(method = "ward.D2")

s1_4 <- s1_4$dist_obj[[1]] %>%
  hclust(method = "ward.D2")

s1_5 <- s1_5$dist_obj[[1]] %>%
  hclust(method = "ward.D2")

s1_6 <- s1_6$dist_obj[[1]] %>%
  hclust(method = "ward.D2")

s1_a <- s1_a$dist_obj[[1]] %>%
  hclust(method = "ward.D2")

s1_3[["labels"]] <- s1_3[["labels"]] %>%
  str_replace_all(c("M07"="","_o"=" 1", "_c"=" 2", "ang"="angry","calm"="calm","disg"="disgust",
                    "exc"="excited","fear"="fear", "hap"="happy",
                    "neut"="neutral","sad"="sad", "surp"="surprise"))
s1_4[["labels"]] <- s1_4[["labels"]] %>%
  str_replace_all(c("M07"="","_o"=" 1", "_c"=" 2", "ang"="angry","calm"="calm","disg"="disgust",
                    "exc"="excited","fear"="fear", "hap"="happy",
                    "neut"="neutral","sad"="sad", "surp"="surprise"))
s1_5[["labels"]] <- s1_5[["labels"]] %>%
  str_replace_all(c("M07"="","_o"=" 1", "_c"=" 2", "ang"="angry","calm"="calm","disg"="disgust",
                    "exc"="excited","fear"="fear", "hap"="happy",
                    "neut"="neutral","sad"="sad", "surp"="surprise"))
s1_6[["labels"]] <- s1_6[["labels"]] %>%
  str_replace_all(c("M07"="","_o"=" 1", "_c"=" 2", "ang"="angry","calm"="calm","disg"="disgust",
                    "exc"="excited","fear"="fear", "hap"="happy",
                    "neut"="neutral","sad"="sad", "surp"="surprise"))
s1_a[["labels"]] <- s1_a[["labels"]] %>%
  str_replace_all(c("M07"="","_o"=" 1", "_c"=" 2", "ang"="angry","calm"="calm","disg"="disgust",
                    "exc"="excited","fear"="fear", "hap"="happy",
                    "neut"="neutral","sad"="sad", "surp"="surprise"))

s1_3_dend <- s1_3 %>% as.dendrogram
s1_4_dend <- s1_4 %>% as.dendrogram
s1_5_dend <- s1_5 %>% as.dendrogram
s1_6_dend <- s1_6 %>% as.dendrogram
s1_a_dend <- s1_a %>% as.dendrogram

# Different Individuals Sort
s2_3 <- avg_dist_byBin %>%
  dplyr::filter(sort=="Sort2"&age_bin=="3 to 4") %>%
  ungroup() %>%
  select(dist_obj)

s2_4 <- avg_dist_byBin %>%
  dplyr::filter(sort=="Sort2"&age_bin=="4 to 5") %>%
  ungroup() %>%
  select(dist_obj)

s2_5 <- avg_dist_byBin %>%
  dplyr::filter(sort=="Sort2"&age_bin=="5 to 6") %>%
  ungroup() %>%
  select(dist_obj)

s2_6 <- avg_dist_byBin %>%
  dplyr::filter(sort=="Sort2"&age_bin=="6 to 7") %>%
  ungroup() %>%
  select(dist_obj)

s2_a <- avg_dist_byBin %>%
  dplyr::filter(sort=="Sort2"&age_bin=="adults") %>%
  ungroup() %>%
  select(dist_obj)

#dist objects
s2_3_dist <- s2_3$dist_obj[[1]]

s2_4_dist <- s2_4$dist_obj[[1]]

s2_5_dist <- s2_5$dist_obj[[1]]

s2_6_dist <- s2_6$dist_obj[[1]]

s2_a_dist <- s2_a$dist_obj[[1]]

#hclust objects
s2_3 <- s2_3$dist_obj[[1]] %>%
  hclust(method = "ward.D2")

s2_4 <- s2_4$dist_obj[[1]] %>%
  hclust(method = "ward.D2")

s2_5 <- s2_5$dist_obj[[1]] %>%
  hclust(method = "ward.D2")

s2_6 <- s2_6$dist_obj[[1]] %>%
  hclust(method = "ward.D2")

s2_a <- s2_a$dist_obj[[1]] %>%
  hclust(method = "ward.D2")

#clean up labels
s2_3[["labels"]] <- s2_3[["labels"]] %>%
  str_replace_all(c("F01"="","F04"="","F07"="","F10"="", "F13"="",
                    "F14"="","F15"="", "F17"="","F22"="","M02"="","M04"="",
                    "M03"="","M05"="","M08"="","M12"="","M14"="","M15"="",
                    "M17"="","_o"=" 1", "_c"=" 2", "ang"="angry", "calm"="calm",
                    "disg"="disgust", "exc"="excited", "fear"="fear",
                    "hap"="happy", "neut"="neutral", "sad"="sad",
                    "surp"="surprise"))
s2_4[["labels"]] <- s2_4[["labels"]] %>%
  str_replace_all(c("F01"="","F04"="","F07"="","F10"="", "F13"="",
                    "F14"="","F15"="", "F17"="","F22"="","M02"="","M04"="",
                    "M03"="","M05"="","M08"="","M12"="","M14"="","M15"="",
                    "M17"="","_o"=" 1", "_c"=" 2", "ang"="angry", "calm"="calm",
                    "disg"="disgust", "exc"="excited", "fear"="fear",
                    "hap"="happy", "neut"="neutral", "sad"="sad",
                    "surp"="surprise"))
s2_5[["labels"]] <- s2_5[["labels"]] %>%
  str_replace_all(c("F01"="","F04"="","F07"="","F10"="", "F13"="",
                    "F14"="","F15"="", "F17"="","F22"="","M02"="","M04"="",
                    "M03"="","M05"="","M08"="","M12"="","M14"="","M15"="",
                    "M17"="","_o"=" 1", "_c"=" 2", "ang"="angry", "calm"="calm",
                    "disg"="disgust", "exc"="excited", "fear"="fear",
                    "hap"="happy", "neut"="neutral", "sad"="sad",
                    "surp"="surprise"))
s2_6[["labels"]] <- s2_6[["labels"]] %>%
  str_replace_all(c("F01"="","F04"="","F07"="","F10"="", "F13"="",
                    "F14"="","F15"="", "F17"="","F22"="","M02"="","M04"="",
                    "M03"="","M05"="","M08"="","M12"="","M14"="","M15"="",
                    "M17"="","_o"=" 1", "_c"=" 2", "ang"="angry", "calm"="calm",
                    "disg"="disgust", "exc"="excited", "fear"="fear",
                    "hap"="happy", "neut"="neutral", "sad"="sad",
                    "surp"="surprise"))
s2_a[["labels"]] <- s2_a[["labels"]] %>%
  str_replace_all(c("F01"="","F04"="","F07"="","F10"="", "F13"="",
                    "F14"="","F15"="", "F17"="","F22"="","M02"="","M04"="",
                    "M03"="","M05"="","M08"="","M12"="","M14"="","M15"="",
                    "M17"="","_o"=" 1", "_c"=" 2", "ang"="angry", "calm"="calm",
                    "disg"="disgust", "exc"="excited", "fear"="fear",
                    "hap"="happy", "neut"="neutral", "sad"="sad",
                    "surp"="surprise"))


s2_3_dend <- s2_3 %>% as.dendrogram
s2_4_dend <- s2_4 %>% as.dendrogram
s2_5_dend <- s2_5 %>% as.dendrogram
s2_6_dend <- s2_6 %>% as.dendrogram
s2_a_dend <- s2_a %>% as.dendrogram
```

## Adjusted Rand

An adjusted Rand index of 0 indicates two clusters have a Rand index that matches the expected value for random groupings (i.e., no similiarity), with higher and lower values indicating higher- or lower-than-chance level similarity between the two clusters.

In all graphs, we are making comparisons between Adults and different groups of children for all possible values of k.

```
# Same Individual: Children versus Adults
m = c(1,2,3,4,5,6,7,8,9,10,11,12,13,14,15,16,17)
calc_AR <- function(x) {
  adj.rand.index(cutree(s1a, k=x),cutree(s1k, k=x))
}
# get adjusted rand for each cluster
ar <- map_dbl(m, calc_AR)
ar <- as.data.frame(ar)
ar <- as_tibble(rownames_to_column(ar))
ar <- ar %>% rename(cluster = rowname, adjusted_rand = ar) %>% mutate(cluster = as.numeric(cluster))
ar_plot1 <- ar %>% ggplot(mapping = aes(x = cluster, y = adjusted_rand))

ar_plot1 <- ar_plot1 + geom_point()+ geom_line(color="red")+
  xlab("k")+
  ylab("Adjusted Rand Index") +
  scale_x_continuous(breaks=c(2,3,4,5,6,7,8,9,10,11,12,13,14,15,16,17), limits=c(2,17)) +
  scale_y_continuous(limits=c(0,1), breaks=c(0,0.1,0.2,0.3,0.4,0.5,0.6,0.7,0.8,0.9,1.0)) +
  theme_linedraw() +
  ggtitle("Same Individual: Children")+
  theme(text=element_text(size=16, family="Times"), plot.title = element_text(size=16, face="bold",family="Times", hjust=.5),
        axis.title.x = element_text(size=20, face="plain",family="Times", vjust=.5),
        axis.title.y = element_text(size=20, face="plain",family="Times", vjust=.5))

#3-year-olds
m = c(1,2,3,4,5,6,7,8,9,10,11,12,13,14,15,16,17)
calc_AR <- function(x) {
  adj.rand.index(cutree(s1a, k=x),cutree(s1_3, k=x))
}
# get adjusted rand for each cluster
ar <- map_dbl(m, calc_AR)
ar <- as.data.frame(ar)
ar <- as_tibble(rownames_to_column(ar))
ar <- ar %>% rename(cluster = rowname, adjusted_rand = ar) %>% mutate(cluster = as.numeric(cluster))
ar_plot1a <- ar %>% ggplot(mapping = aes(x = cluster, y = adjusted_rand))

ar_plot1a <- ar_plot1a + geom_point()+ geom_line(color="red")+
  xlab("k")+
  ylab("Adjusted Rand Index") +
  scale_x_continuous(breaks=c(2,3,4,5,6,7,8,9,10,11,12,13,14,15,16,17), limits=c(2,17)) +
  scale_y_continuous(limits=c(0,1), breaks=c(0,0.1,0.2,0.3,0.4,0.5,0.6,0.7,0.8,0.9,1.0)) +
  theme_linedraw() +
  ggtitle("3-year-olds")+
  theme(text=element_text(size=16, family="Times"), plot.title = element_text(size=16, face="bold",family="Times", hjust=.5),
        axis.title.x = element_text(size=14, face="plain",family="Times", vjust=.5),
        axis.title.y = element_text(size=14, face="plain",family="Times", vjust=.5))

#4-year-olds
m = c(1,2,3,4,5,6,7,8,9,10,11,12,13,14,15,16,17)
calc_AR <- function(x) {
  adj.rand.index(cutree(s1a, k=x),cutree(s1_4, k=x))
}
# get adjusted rand for each cluster
ar <- map_dbl(m, calc_AR)
ar <- as.data.frame(ar)
ar <- as_tibble(rownames_to_column(ar))
ar <- ar %>% rename(cluster = rowname, adjusted_rand = ar) %>% mutate(cluster = as.numeric(cluster))
ar_plot1b <- ar %>% ggplot(mapping = aes(x = cluster, y = adjusted_rand))

ar_plot1b <- ar_plot1b + geom_point()+ geom_line(color="red")+
  xlab("k")+
  ylab("Adjusted Rand Index") +
  scale_x_continuous(breaks=c(2,3,4,5,6,7,8,9,10,11,12,13,14,15,16,17), limits=c(2,17)) +
  scale_y_continuous(limits=c(0,1), breaks=c(0,0.1,0.2,0.3,0.4,0.5,0.6,0.7,0.8,0.9,1.0)) +
  theme_linedraw() +
  ggtitle("4-year-olds")+
  theme(text=element_text(size=16, family="Times"), plot.title = element_text(size=16, face="bold",family="Times", hjust=.5),
        axis.title.x = element_text(size=14, face="plain",family="Times", vjust=.5),
        axis.title.y = element_text(size=14, face="plain",family="Times", vjust=.5))

#5-year-olds
m = c(1,2,3,4,5,6,7,8,9,10,11,12,13,14,15,16,17)
calc_AR <- function(x) {
  adj.rand.index(cutree(s1a, k=x),cutree(s1_5, k=x))
}
# get adjusted rand for each cluster
ar <- map_dbl(m, calc_AR)
ar <- as.data.frame(ar)
ar <- as_tibble(rownames_to_column(ar))
ar <- ar %>% rename(cluster = rowname, adjusted_rand = ar) %>% mutate(cluster = as.numeric(cluster))
ar_plot1c <- ar %>% ggplot(mapping = aes(x = cluster, y = adjusted_rand))

ar_plot1c <- ar_plot1c + geom_point()+ geom_line(color="red")+
  xlab("k")+
  ylab("Adjusted Rand Index") +
  scale_x_continuous(breaks=c(2,3,4,5,6,7,8,9,10,11,12,13,14,15,16,17), limits=c(2,17)) +
  scale_y_continuous(limits=c(0,1), breaks=c(0,0.1,0.2,0.3,0.4,0.5,0.6,0.7,0.8,0.9,1.0)) +
  theme_linedraw() +
  ggtitle("5-year-olds")+
  theme(text=element_text(size=16, family="Times"), plot.title = element_text(size=16, face="bold",family="Times", hjust=.5),
        axis.title.x = element_text(size=14, face="plain",family="Times", vjust=.5),
        axis.title.y = element_text(size=14, face="plain",family="Times", vjust=.5))

#6-year-olds
m = c(1,2,3,4,5,6,7,8,9,10,11,12,13,14,15,16,17)
calc_AR <- function(x) {
  adj.rand.index(cutree(s1a, k=x),cutree(s1_6, k=x))
}
# get adjusted rand for each cluster
ar <- map_dbl(m, calc_AR)
ar <- as.data.frame(ar)
ar <- as_tibble(rownames_to_column(ar))
ar <- ar %>% rename(cluster = rowname, adjusted_rand = ar) %>% mutate(cluster = as.numeric(cluster))
ar_plot1d <- ar %>% ggplot(mapping = aes(x = cluster, y = adjusted_rand))

ar_plot1d <- ar_plot1d + geom_point()+ geom_line(color="red")+
  xlab("k")+
  ylab("Adjusted Rand Index") +
  scale_x_continuous(breaks=c(2,3,4,5,6,7,8,9,10,11,12,13,14,15,16,17), limits=c(2,17)) +
  scale_y_continuous(limits=c(0,1), breaks=c(0,0.1,0.2,0.3,0.4,0.5,0.6,0.7,0.8,0.9,1.0)) +
  theme_linedraw() +
  ggtitle("6-year-olds")+
  theme(text=element_text(size=16, family="Times"), plot.title = element_text(size=16, face="bold",family="Times", hjust=.5),
        axis.title.x = element_text(size=14, face="plain",family="Times", vjust=.5),
        axis.title.y = element_text(size=14, face="plain",family="Times", vjust=.5))

# Diff Individual: Children versus Adults
calc_AR <- function(x) {
  adj.rand.index(cutree(s2a, k=x),cutree(s2k, k=x))
}

ar <- map_dbl(m, calc_AR)
ar <- as.data.frame(ar)
ar <- as_tibble(rownames_to_column(ar))
ar <- ar %>% rename(cluster = rowname, adjusted_rand = ar) %>% mutate(cluster = as.numeric(cluster))
ar_plot2 <- ar %>% ggplot(mapping = aes(x = cluster, y = adjusted_rand))

ar_plot2 <- ar_plot2 + geom_point()+ geom_line(color="red")+
  xlab("k")+
  ylab("Adjusted Rand Index") +
  scale_x_continuous(breaks=c(2,3,4,5,6,7,8,9,10,11,12,13,14,15,16,17), limits=c(2,17)) +
  scale_y_continuous(limits=c(0,1), breaks=c(0,0.1,0.2,0.3,0.4,0.5,0.6,0.7,0.8,0.9,1.0)) +
  theme_linedraw() +
  ggtitle("Diff. Individuals: Children")+
  theme(text=element_text(size=16, family="Times"), plot.title = element_text(size=16, face="bold",family="Times", hjust=.5),
        axis.title.x = element_text(size=20, face="plain",family="Times", vjust=.5),
        axis.title.y = element_text(size=20, face="plain",family="Times", vjust=.5))

#3-year-olds
m = c(1,2,3,4,5,6,7,8,9,10,11,12,13,14,15,16,17)
calc_AR <- function(x) {
  adj.rand.index(cutree(s2a, k=x),cutree(s2_3, k=x))
}
# get adjusted rand for each cluster
ar <- map_dbl(m, calc_AR)
ar <- as.data.frame(ar)
ar <- as_tibble(rownames_to_column(ar))
ar <- ar %>% rename(cluster = rowname, adjusted_rand = ar) %>% mutate(cluster = as.numeric(cluster))
ar_plot2a <- ar %>% ggplot(mapping = aes(x = cluster, y = adjusted_rand))

ar_plot2a <- ar_plot2a + geom_point()+ geom_line(color="red")+
  xlab("k")+
  ylab("Adjusted Rand Index") +
  scale_x_continuous(breaks=c(2,3,4,5,6,7,8,9,10,11,12,13,14,15,16,17), limits=c(2,17)) +
  scale_y_continuous(limits=c(0,1), breaks=c(0,0.1,0.2,0.3,0.4,0.5,0.6,0.7,0.8,0.9,1.0)) +
  theme_linedraw() +
  ggtitle("3-year-olds")+
  theme(text=element_text(size=16, family="Times"), plot.title = element_text(size=16, face="bold",family="Times", hjust=.5),
        axis.title.x = element_text(size=14, face="plain",family="Times", vjust=.5),
        axis.title.y = element_text(size=14, face="plain",family="Times", vjust=.5))

#4-year-olds
m = c(1,2,3,4,5,6,7,8,9,10,11,12,13,14,15,16,17)
calc_AR <- function(x) {
  adj.rand.index(cutree(s2a, k=x),cutree(s2_4, k=x))
}
# get adjusted rand for each cluster
ar <- map_dbl(m, calc_AR)
ar <- as.data.frame(ar)
ar <- as_tibble(rownames_to_column(ar))
ar <- ar %>% rename(cluster = rowname, adjusted_rand = ar) %>% mutate(cluster = as.numeric(cluster))
ar_plot2b <- ar %>% ggplot(mapping = aes(x = cluster, y = adjusted_rand))

ar_plot2b <- ar_plot2b + geom_point()+ geom_line(color="red")+
  xlab("k")+
  ylab("Adjusted Rand Index") +
  scale_x_continuous(breaks=c(2,3,4,5,6,7,8,9,10,11,12,13,14,15,16,17), limits=c(2,17)) +
  scale_y_continuous(limits=c(0,1), breaks=c(0,0.1,0.2,0.3,0.4,0.5,0.6,0.7,0.8,0.9,1.0)) +
  theme_linedraw() +
  ggtitle("4-year-olds")+
  theme(text=element_text(size=16, family="Times"), plot.title = element_text(size=16, face="bold",family="Times", hjust=.5),
        axis.title.x = element_text(size=14, face="plain",family="Times", vjust=.5),
        axis.title.y = element_text(size=14, face="plain",family="Times", vjust=.5))

#5-year-olds
m = c(1,2,3,4,5,6,7,8,9,10,11,12,13,14,15,16,17)
calc_AR <- function(x) {
  adj.rand.index(cutree(s2a, k=x),cutree(s2_5, k=x))
}
# get adjusted rand for each cluster
ar <- map_dbl(m, calc_AR)
ar <- as.data.frame(ar)
ar <- as_tibble(rownames_to_column(ar))
ar <- ar %>% rename(cluster = rowname, adjusted_rand = ar) %>% mutate(cluster = as.numeric(cluster))
ar_plot2c <- ar %>% ggplot(mapping = aes(x = cluster, y = adjusted_rand))

ar_plot2c <- ar_plot2c + geom_point()+ geom_line(color="red")+
  xlab("k")+
  ylab("Adjusted Rand Index") +
  scale_x_continuous(breaks=c(2,3,4,5,6,7,8,9,10,11,12,13,14,15,16,17), limits=c(2,17)) +
  scale_y_continuous(limits=c(0,1), breaks=c(0,0.1,0.2,0.3,0.4,0.5,0.6,0.7,0.8,0.9,1.0)) +
  theme_linedraw() +
  ggtitle("5-year-olds")+
  theme(text=element_text(size=16, family="Times"), plot.title = element_text(size=16, face="bold",family="Times", hjust=.5),
        axis.title.x = element_text(size=14, face="plain",family="Times", vjust=.5),
        axis.title.y = element_text(size=14, face="plain",family="Times", vjust=.5))

#6-year-olds
m = c(1,2,3,4,5,6,7,8,9,10,11,12,13,14,15,16,17)
calc_AR <- function(x) {
  adj.rand.index(cutree(s2a, k=x),cutree(s2_6, k=x))
}
# get adjusted rand for each cluster
ar <- map_dbl(m, calc_AR)
ar <- as.data.frame(ar)
ar <- as_tibble(rownames_to_column(ar))
ar <- ar %>% rename(cluster = rowname, adjusted_rand = ar) %>% mutate(cluster = as.numeric(cluster))
ar_plot2d <- ar %>% ggplot(mapping = aes(x = cluster, y = adjusted_rand))

ar_plot2d <- ar_plot2d + geom_point()+ geom_line(color="red")+
  xlab("k")+
  ylab("Adjusted Rand Index") +
  scale_x_continuous(breaks=c(2,3,4,5,6,7,8,9,10,11,12,13,14,15,16,17), limits=c(2,17)) +
  scale_y_continuous(limits=c(0,1), breaks=c(0,0.1,0.2,0.3,0.4,0.5,0.6,0.7,0.8,0.9,1.0)) +
  theme_linedraw() +
  ggtitle("6-year-olds")+
  theme(text=element_text(size=16, family="Times"), plot.title = element_text(size=16, face="bold",family="Times", hjust=.5),
        axis.title.x = element_text(size=14, face="plain",family="Times", vjust=.5),
        axis.title.y = element_text(size=14, face="plain",family="Times", vjust=.5))


# All Children
plot_grid(ar_plot1,ar_plot2)
```

```
# Same Individual Sort
title1 <- ggdraw() +
  draw_label("Same Individual Sort",
    fontface = 'bold',
    x = 0,
    hjust = 0) +
  theme(plot.margin = margin(0, 0, 0, 7))
plot_row1 <- plot_grid(ar_plot1a,ar_plot1b,ar_plot1c,ar_plot1d)
plot_grid(
  title1, plot_row1,
  ncol = 1,
  rel_heights = c(0.1, 1)
)
```

```
# Different Individuals Sort
title2 <- ggdraw() +
  draw_label("Diff. Individuals Sort",
    fontface = 'bold',
    x = 0,
    hjust = 0) +
  theme(plot.margin = margin(0, 0, 0, 7))
plot_row2 <- plot_grid(ar_plot2a,ar_plot2b,ar_plot2c,ar_plot2d)
plot_grid(
  title2, plot_row2,
  ncol = 1,
  rel_heights = c(0.1, 1)
)
```

## FM Index

The Fowlkes-Mallows (FM) Index measures the similarity between two hierarchical clusterings (Fowlkes & Mallow, 1983). It ranges from 0 (no similarity) to 1 (perfect similarity). The index aims to capture how likely it is that two clusters contain the same faces. Below we plot the FM-Index for all possible cluster solutions. Within this plot, the dashed black line indicates the null hypothesis (the similarity expected if the two dendrograms are “not similar”), the red line is the rejection line, above which we can say that the two dendrograms are similar, and the dotted black line is the FM-Index that we observe when comparing the children’s hierarchical clustering solution to that of adults. In all graphs, we are making comparisons between Adults and different groups of children.

```
# Same Individual Sort:
par(mfrow = c(2,3))
Bk_plot(s1k, s1a, main = "Same Individual Sort \nAll Children")
Bk_plot(s1a, s1_3, main = "Same Individual Sort \n3-year-olds")
Bk_plot(s1a, s1_4, main = "Same Individual Sort \n4-year-olds")
Bk_plot(s1a, s1_5, main = "Same Individual Sort \n5-year-olds")
Bk_plot(s1a, s1_6, main = "Same Individual Sort \n6-year-olds")

# Different Individuals Sort
par(mfrow = c(2,3))
```

```
Bk_plot(s2a, s2k, main = "Diff. Individual Sort \nAll Children")
Bk_plot(s2a, s2_3, main = "Diff. Individual Sort \n3-year-olds")
Bk_plot(s2a, s2_4, main = "Diff. Individual Sort \n4-year-olds")
Bk_plot(s2a, s2_5, main = "Diff. Individual Sort \n5-year-olds")
Bk_plot(s2a, s2_6, main = "Diff. Individual Sort \n6-year-olds")
```

## Entanglement

Entanglement measures whether the labels at the bottom of two dendrograms are aligned with one another. A value of 0 indicates perfect alignment, while a value of 1 indicates no alignment. To optimize alignment, we first used the untangle method. For all values, we are making comparisons between Adults and different groups of children.

```
library(gt)
tang_tracker1 <- tibble(Age_Group = character(), entanglement= numeric())
tang_tracker2 <- tibble(Age_Group = character(), entanglement= numeric())

# Same Individual Sort
set.seed(51314)

p0 <- dendlist("Adults" = s1_a_dend, "Children" = s1k_dend)
p1 <- dendlist("Adults" = s1_a_dend, "Kids" = s1_3_dend)
p2 <- dendlist("Adults" = s1_a_dend, "Kids" = s1_4_dend)
p3 <- dendlist("Adults" = s1_a_dend, "Kids" = s1_5_dend)
p4 <- dendlist("Adults" = s1_a_dend, "Kids" = s1_6_dend)

x_untangle0 <- p0 %>% untangle(method = "random", R=1000)
x_untangle1 <- p1 %>% untangle(method = "random", R=1000)
x_untangle2 <- p2 %>% untangle(method = "random", R=1000)
x_untangle3 <- p3 %>% untangle(method = "random", R=1000)
x_untangle4 <- p4 %>% untangle(method = "random", R=1000)

tang_tracker1 <- bind_rows(tang_tracker1, tibble(Age_Group = "All Children", entanglement=entanglement(x_untangle0)))
tang_tracker1 <- bind_rows(tang_tracker1, tibble(Age_Group = "3-year-olds", entanglement=entanglement(x_untangle1)))
tang_tracker1 <- bind_rows(tang_tracker1, tibble(Age_Group = "4-year-olds", entanglement=entanglement(x_untangle2)))
tang_tracker1 <- bind_rows(tang_tracker1, tibble(Age_Group = "5-year-olds", entanglement=entanglement(x_untangle3)))
tang_tracker1 <- bind_rows(tang_tracker1, tibble(Age_Group = "6-year-olds", entanglement=entanglement(x_untangle4)))


# Different Individuals Sort
set.seed(51314)

p0 <- dendlist("Adults" = s2_a_dend, "Children" = s2k_dend)
p1 <- dendlist("Adults" = s2_a_dend, "Kids" = s2_3_dend)
p2 <- dendlist("Adults" = s2_a_dend, "Kids" = s2_4_dend)
p3 <- dendlist("Adults" = s2_a_dend, "Kids" = s2_5_dend)
p4 <- dendlist("Adults" = s2_a_dend, "Kids" = s2_6_dend)

x_untangle0 <- p0 %>% untangle(method = "random", R=1000)
x_untangle1 <- p1 %>% untangle(method = "random", R=1000)
x_untangle2 <- p2 %>% untangle(method = "random", R=1000)
x_untangle3 <- p3 %>% untangle(method = "random", R=1000)
x_untangle4 <- p4 %>% untangle(method = "random", R=1000)

tang_tracker2 <- bind_rows(tang_tracker2, tibble(Age_Group = "All Children", entanglement=entanglement(x_untangle0)))
tang_tracker2 <- bind_rows(tang_tracker2, tibble(Age_Group = "3-year-olds", entanglement=entanglement(x_untangle1)))
tang_tracker2 <- bind_rows(tang_tracker2, tibble(Age_Group = "4-year-olds", entanglement=entanglement(x_untangle2)))
tang_tracker2 <- bind_rows(tang_tracker2, tibble(Age_Group = "5-year-olds", entanglement=entanglement(x_untangle3)))
tang_tracker2 <- bind_rows(tang_tracker2, tibble(Age_Group = "6-year-olds", entanglement=entanglement(x_untangle4)))

tab1 <- bind_cols(tang_tracker1, tang_tracker2)
tab1 %>% gt(tab1) %>% cols_align(align="center") %>% cols_label(Age_Group...1 = "Age Groups", entanglement...2=md("Entanglement"),Age_Group...3 = "Age Groups", entanglement...4=md("Entanglement")) %>%   tab_header(
    title = md("Entanglement"),
    subtitle = md("**Same Individual versus Different Individuals Sort**")
  )
```

| Entanglement | | | |
| --- | --- | --- | --- |
| **Same Individual versus Different Individuals Sort** | | | |
| Age Groups | Entanglement | Age Groups | Entanglement |
| --- | --- | --- | --- |
| All Children | 0.05047748 | All Children | 0.07209138 |
| 3-year-olds | 0.33038003 | 3-year-olds | 0.22913182 |
| 4-year-olds | 0.14727156 | 4-year-olds | 0.16351788 |
| 5-year-olds | 0.09630867 | 5-year-olds | 0.08110832 |
| 6-year-olds | 0.07269705 | 6-year-olds | 0.09232746 |

## Cophenetic Correlation

c is the cophenetic correlation coefficient between the two dendrograms. This value ranges from -1 to 1 with values near 0 suggesting that the two dendrograms are not statistically similar. For all values, we are making comparisons between Adults and different groups of children.

```
cor_tracker1 <- tibble(Age_Group = character(),c = numeric())
cor_tracker2 <- tibble(Age_Group = character(),c = numeric())

#Same Individual Sort (Comparison to Adults)
cor_tracker1 <- bind_rows(cor_tracker1, tibble(Age_Group = "All Children", c=cor_cophenetic(s1a, s1k)))
cor_tracker1 <- bind_rows(cor_tracker1, tibble(Age_Group = "3-year-olds", c=cor_cophenetic(s1a, s1_3)))
cor_tracker1 <- bind_rows(cor_tracker1, tibble(Age_Group = "4-year-olds", c=cor_cophenetic(s1a, s1_4)))
cor_tracker1 <- bind_rows(cor_tracker1, tibble(Age_Group = "5-year-olds", c=cor_cophenetic(s1a, s1_5)))
cor_tracker1 <- bind_rows(cor_tracker1, tibble(Age_Group = "6-year-olds", c=cor_cophenetic(s1a, s1_6)))

#Different Individuals Sort (Comparison to Adults)
cor_tracker2 <- bind_rows(cor_tracker2, tibble(Age_Group = "All Children", c=cor_cophenetic(s2a, s2k)))
cor_tracker2 <- bind_rows(cor_tracker2, tibble(Age_Group = "3-year-olds", c=cor_cophenetic(s2a, s2_3)))
cor_tracker2 <- bind_rows(cor_tracker2, tibble(Age_Group = "4-year-olds", c=cor_cophenetic(s2a, s2_4)))
cor_tracker2 <- bind_rows(cor_tracker2, tibble(Age_Group = "5-year-olds", c=cor_cophenetic(s2a, s2_5)))
cor_tracker2 <- bind_rows(cor_tracker2, tibble(Age_Group = "6-year-olds", c=cor_cophenetic(s2a, s2_6)))

tab1 <- bind_cols(cor_tracker1, cor_tracker2)
tab1 %>% gt(tab1) %>% cols_align(align="center") %>% cols_label(Age_Group...1 = "Age Groups", c...2=md("*c*"),Age_Group...3 = "Age Groups", c...4=md("*c*")) %>%   tab_header(
    title = md("Cophenetic Correlation"),
    subtitle = md("**Same Individual versus Different Individuals Sort**")
  )
```

| Cophenetic Correlation | | | |
| --- | --- | --- | --- |
| **Same Individual versus Different Individuals Sort** | | | |
| Age Groups | *c* | Age Groups | *c* |
| --- | --- | --- | --- |
| All Children | 0.5171840210 | All Children | 0.4047446 |
| 3-year-olds | 0.0002808651 | 3-year-olds | 0.2061645 |
| 4-year-olds | 0.1953945055 | 4-year-olds | 0.2005345 |
| 5-year-olds | 0.4087643625 | 5-year-olds | 0.3779366 |
| 6-year-olds | 0.6486572036 | 6-year-olds | 0.4021863 |

## Baker’s Gamma

Baker’s Gamma Index is a measure of similarity between two dendrograms. This value ranges from -1 to 1 with values near 0 suggesting that the two dendrograms are not statistically similar. For all values, we are making comparisons between Adults and different groups of children.

```
cor_tracker1 <- tibble(Age_Group = character(), gamma = numeric())
cor_tracker2 <- tibble(Age_Group = character(), gamma = numeric())

#Same Individual Sort (Comparison to Adults)
cor_tracker1 <- bind_rows(cor_tracker1, tibble(Age_Group = "All Children", gamma=cor_bakers_gamma(s1a, s1k)))
cor_tracker1 <- bind_rows(cor_tracker1, tibble(Age_Group = "3-year-olds", gamma=cor_bakers_gamma(s1a, s1_3)))
cor_tracker1 <- bind_rows(cor_tracker1, tibble(Age_Group = "4-year-olds", gamma=cor_bakers_gamma(s1a, s1_4)))
cor_tracker1 <- bind_rows(cor_tracker1, tibble(Age_Group = "5-year-olds", gamma=cor_bakers_gamma(s1a, s1_5)))
cor_tracker1 <- bind_rows(cor_tracker1, tibble(Age_Group = "6-year-olds", gamma=cor_bakers_gamma(s1a, s1_6)))

#Different Individuals Sort (Comparison to Adults)
cor_tracker2 <- bind_rows(cor_tracker2, tibble(Age_Group = "All Children", gamma=cor_bakers_gamma(s2a, s2k)))
cor_tracker2 <- bind_rows(cor_tracker2, tibble(Age_Group = "3-year-olds", gamma=cor_bakers_gamma(s2a, s2_3)))
cor_tracker2 <- bind_rows(cor_tracker2, tibble(Age_Group = "4-year-olds", gamma=cor_bakers_gamma(s2a, s2_4)))
cor_tracker2 <- bind_rows(cor_tracker2, tibble(Age_Group = "5-year-olds", gamma=cor_bakers_gamma(s2a, s2_5)))
cor_tracker2 <- bind_rows(cor_tracker2, tibble(Age_Group = "6-year-olds", gamma=cor_bakers_gamma(s2a, s2_6)))

tab1 <- bind_cols(cor_tracker1, cor_tracker2)
tab1 %>% gt(tab1) %>% cols_align(align="center") %>% cols_label(Age_Group...1 = "Age Groups", gamma...2=md("Baker's Gamma"),Age_Group...3 = "Age Groups", gamma...4=md("Baker's Gamma")) %>%   tab_header(
    title = md("Baker's Gamma"),
    subtitle = md("**Same Individual versus Different Individuals Sort**")
  )
```

| Baker's Gamma | | | |
| --- | --- | --- | --- |
| **Same Individual versus Different Individuals Sort** | | | |
| Age Groups | Baker's Gamma | Age Groups | Baker's Gamma |
| --- | --- | --- | --- |
| All Children | 0.50154886 | All Children | 0.3315111 |
| 3-year-olds | -0.01084084 | 3-year-olds | 0.1733218 |
| 4-year-olds | 0.15683565 | 4-year-olds | 0.1850167 |
| 5-year-olds | 0.43391704 | 5-year-olds | 0.3564704 |
| 6-year-olds | 0.50715301 | 6-year-olds | 0.3030421 |

# 6c. Hierarchical Clustering: Selecting method and k

## Method: Same Individual Sort

To select our hierarchical clustering method (average, single, complete, or Ward’s), we analyzed the agglomerative coefficient (AC). AC gives a measure of the strength of the clustering structure, and we found that Ward’s method gave the best solution. Furthermore, Ward’s is a generally preferred method for agglomerative (bottom-up) hierarchical clustering (Kaufman & Rousseeuw, 2009; Boehmke & Greenwell, 2020).

```
#methods
m <- c( "average", "single", "complete", "ward")

# Different Age Groups
names(m) <- c( "average", "single", "complete", "ward")
ac <- function(x) {
  agnes(s1a_dist, method = x)$ac
}
t1 <- as.data.frame(map_dbl(m, ac)) %>% rownames_to_column() %>% rename(method="rowname",AC=`map_dbl(m, ac)`)

names(m) <- c( "average", "single", "complete", "ward")
ac <- function(x) {
  agnes(s1k_dist, method = x)$ac
}
t2 <- as.data.frame(map_dbl(m, ac)) %>% rownames_to_column() %>% rename(method="rowname",AC=`map_dbl(m, ac)`)

names(m) <- c( "average", "single", "complete", "ward")
ac <- function(x) {
  agnes(s1_3_dist, method = x)$ac
}
t3 <- as.data.frame(map_dbl(m, ac)) %>% rownames_to_column() %>% rename(method="rowname",AC=`map_dbl(m, ac)`)

names(m) <- c( "average", "single", "complete", "ward")
ac <- function(x) {
  agnes(s1_4_dist, method = x)$ac
}
t4 <- as.data.frame(map_dbl(m, ac)) %>% rownames_to_column() %>% rename(method="rowname",AC=`map_dbl(m, ac)`)

names(m) <- c( "average", "single", "complete", "ward")
ac <- function(x) {
  agnes(s1_5_dist, method = x)$ac
}
t5 <- as.data.frame(map_dbl(m, ac)) %>% rownames_to_column() %>% rename(method="rowname",AC=`map_dbl(m, ac)`)

names(m) <- c( "average", "single", "complete", "ward")
ac <- function(x) {
  agnes(s1_6_dist, method = x)$ac
}
t6 <- as.data.frame(map_dbl(m, ac)) %>% rownames_to_column() %>% rename(method="rowname",AC=`map_dbl(m, ac)`)

tab1 <- bind_cols(t1,t2,t3,t4,t5,t6)
gt(tab1) %>% cols_align(align="center") %>% tab_header(title = md("Selecting Hierarchical Clustering Method"), subtitle = md("**Same Individual Sort**"))%>% cols_align(align="center") %>% cols_label(method...1 = "Adults", AC...2=md("*AC*"), method...3 = "Children", AC...4=md("*AC*"),method...5 = "3-years",
AC...6=md("*AC*"),method...7 = "4-years", 
AC...8=md("*AC*"),method...9 = "5-years", AC...10=md("*AC*"),method...11 = "6-years", 
AC...12=md("*AC*"))
```

| Selecting Hierarchical Clustering Method | | | | | | | | | | | |
| --- | --- | --- | --- | --- | --- | --- | --- | --- | --- | --- | --- |
| **Same Individual Sort** | | | | | | | | | | | |
| Adults | *AC* | Children | *AC* | 3-years | *AC* | 4-years | *AC* | 5-years | *AC* | 6-years | *AC* |
| --- | --- | --- | --- | --- | --- | --- | --- | --- | --- | --- | --- |
| average | 0.5742989 | average | 0.2425198 | average | 0.2123615 | average | 0.2033784 | average | 0.3628579 | average | 0.4149369 |
| single | 0.3604857 | single | 0.1409394 | single | 0.1605144 | single | 0.1285547 | single | 0.2462822 | single | 0.2783900 |
| complete | 0.6666816 | complete | 0.3096061 | complete | 0.3318593 | complete | 0.2778497 | complete | 0.4834586 | complete | 0.5055202 |
| ward | 0.8180321 | ward | 0.5323046 | ward | 0.4088731 | ward | 0.4066864 | ward | 0.6732763 | ward | 0.6974837 |

## Method: Different Individual Sort

To select our hierarchical clustering method (average, single, complete, or Ward’s), we analyzed the agglomerative coefficient (AC). AC gives a measure of the strength of the clustering structure, and we found that Ward’s method gave the best solution. Furthermore, Ward’s is a generally preferred method for agglomerative (bottom-up) hierarchical clustering (Kaufman & Rousseeuw, 2009; Boehmke & Greenwell, 2020).

```
#methods
m <- c( "average", "single", "complete", "ward")

# Different Age Groups
names(m) <- c( "average", "single", "complete", "ward")
ac <- function(x) {
  agnes(s2a_dist, method = x)$ac
}
t1 <- as.data.frame(map_dbl(m, ac)) %>% rownames_to_column() %>% rename(method="rowname",AC=`map_dbl(m, ac)`)

names(m) <- c( "average", "single", "complete", "ward")
ac <- function(x) {
  agnes(s2k_dist, method = x)$ac
}
t2 <- as.data.frame(map_dbl(m, ac)) %>% rownames_to_column() %>% rename(method="rowname",AC=`map_dbl(m, ac)`)

names(m) <- c( "average", "single", "complete", "ward")
ac <- function(x) {
  agnes(s2_3_dist, method = x)$ac
}
t3 <- as.data.frame(map_dbl(m, ac)) %>% rownames_to_column() %>% rename(method="rowname",AC=`map_dbl(m, ac)`)

names(m) <- c( "average", "single", "complete", "ward")
ac <- function(x) {
  agnes(s2_4_dist, method = x)$ac
}
t4 <- as.data.frame(map_dbl(m, ac)) %>% rownames_to_column() %>% rename(method="rowname",AC=`map_dbl(m, ac)`)

names(m) <- c( "average", "single", "complete", "ward")
ac <- function(x) {
  agnes(s2_5_dist, method = x)$ac
}
t5 <- as.data.frame(map_dbl(m, ac)) %>% rownames_to_column() %>% rename(method="rowname",AC=`map_dbl(m, ac)`)

names(m) <- c( "average", "single", "complete", "ward")
ac <- function(x) {
  agnes(s2_6_dist, method = x)$ac
}
t6 <- as.data.frame(map_dbl(m, ac)) %>% rownames_to_column() %>% rename(method="rowname",AC=`map_dbl(m, ac)`)

tab1 <- bind_cols(t1,t2,t3,t4,t5,t6)
gt(tab1) %>% cols_align(align="center") %>% tab_header(title = md("Selecting Hierarchical Clustering Method"), subtitle = md("**Different Individuals Sort**"))%>% cols_align(align="center") %>% cols_label(method...1 = "Adults", AC...2=md("*AC*"), method...3 = "Children", AC...4=md("*AC*"),method...5 = "3-years",
AC...6=md("*AC*"),method...7 = "4-years", 
AC...8=md("*AC*"),method...9 = "5-years", AC...10=md("*AC*"),method...11 = "6-years", 
AC...12=md("*AC*"))
```

| Selecting Hierarchical Clustering Method | | | | | | | | | | | |
| --- | --- | --- | --- | --- | --- | --- | --- | --- | --- | --- | --- |
| **Different Individuals Sort** | | | | | | | | | | | |
| Adults | *AC* | Children | *AC* | 3-years | *AC* | 4-years | *AC* | 5-years | *AC* | 6-years | *AC* |
| --- | --- | --- | --- | --- | --- | --- | --- | --- | --- | --- | --- |
| average | 0.6016824 | average | 0.2309338 | average | 0.2029392 | average | 0.1987919 | average | 0.3239638 | average | 0.4360385 |
| single | 0.3679243 | single | 0.1302087 | single | 0.1168117 | single | 0.1429537 | single | 0.1914711 | single | 0.2365958 |
| complete | 0.6788049 | complete | 0.2897238 | complete | 0.3057533 | complete | 0.2763106 | complete | 0.4317873 | complete | 0.5173554 |
| ward | 0.8192563 | ward | 0.5254612 | ward | 0.3898247 | ward | 0.4152076 | ward | 0.6101728 | ward | 0.7085441 |

## Selecting k: Same Individual Sort

As we used hierarchical clustering and presented dendrograms, we displayed all possible clusters in the manuscript. However, we did chose to highlight the three cluster solution (k=3) in the dendrograms we displayed. Below we present bar plots of the height of the different clusters in the dendrogram. Large changes in these heights can be helpful for determining the optimal number of clusters to display. Through observations of these graphs, subjective evaluation of the dendrograms, patterns of valence among the images, and a desire to be consistent across different age ranges, we opted to display the three cluster solution. However, for full transparency we also report similarity indices for all values of k in section 6b (above).

```
s1_cluster <- as.data.frame(s1a$height) %>% rename(adults="s1a$height") %>% mutate(children=s1k$height,three_years=s1_3$height,four_years=s1_4$height,five_years=s1_5$height,six_years=s1_6$height) %>% rownames_to_column() %>% rename(cluster=rowname) %>% mutate(cluster=abs(as.numeric(cluster)-18))

s1_cluster_dL <- s1_cluster %>% group_by(cluster) %>% gather(key = age_group, value = height, adults,children,three_years,four_years,five_years,six_years)

s1_cluster_dL <- s1_cluster_dL %>% mutate(age_group=case_when(
  age_group == "adults" ~ "adults",
  age_group =="children" ~ "children",
  age_group =="three_years" ~ "3-year-olds",
  age_group =="four_years" ~ "4-year-olds",
  age_group =="five_years" ~ "5-year-olds",
  age_group =="six_years" ~ "6-year-olds"))

ggplot(data=s1_cluster_dL,aes(x=cluster,y=height, fill=age_group)) + geom_bar(stat='identity', color="black") + facet_wrap(~age_group) +
  theme(legend.position="none")+
  scale_x_continuous(breaks=c(1,3,5,7,9,11,13,15,17))+
  ggtitle("Height of dendrogram for cluster k")
```

## Selecting k: Different Individuals Sort

As we used hierarchical clustering and presented dendrograms, we displayed all possible clusters in the manuscript. However, we did chose to highlight the three cluster solution (k=3) in the dendrograms we displayed. Below we present bar plots of the height of the different clusters in the dendrogram. Large changes in these heights can be helpful for determining the optimal number of clusters to display. Through observations of these graphs, subjective evaluation of the dendrograms, patterns of valence among the images, and a desire to be consistent across different age ranges, we opted to display the three cluster solution. However, for full transparency we also report similarity indices for all values of k in section 6b (above).

```
s2_cluster <- as.data.frame(s2a$height) %>% rename(adults="s2a$height") %>% mutate(children=s2k$height,three_years=s2_3$height,four_years=s2_4$height,five_years=s2_5$height,six_years=s2_6$height) %>% rownames_to_column() %>% rename(cluster=rowname) %>% mutate(cluster=abs(as.numeric(cluster)-18))

s2_cluster_dL <- s2_cluster %>% group_by(cluster) %>% gather(key = age_group, value = height, adults,children,three_years,four_years,five_years,six_years)

s2_cluster_dL <- s2_cluster_dL %>% mutate(age_group=case_when(
  age_group == "adults" ~ "adults",
  age_group =="children" ~ "children",
  age_group =="three_years" ~ "3-year-olds",
  age_group =="four_years" ~ "4-year-olds",
  age_group =="five_years" ~ "5-year-olds",
  age_group =="six_years" ~ "6-year-olds"))

ggplot(data=s2_cluster_dL,aes(x=cluster,y=height, fill=age_group)) + geom_bar(stat='identity', color="black") + facet_wrap(~age_group) +
  theme(legend.position="none") +
  scale_x_continuous(breaks=c(1,3,5,7,9,11,13,15,17))+
  ggtitle("Height of dendrogram for cluster k")
```

# 7. Rating Data

Here we present more information about the ratings for all of the different images. Valence and arousal were rated on 7-point Likert scales. Positivity and Negativity where rated on a 4-point scale using the evaluative space grid.

```
library(gmodels)
raw_ratings <- read.csv(here(root_path,"analysis","paper_2020","processed_data","ratings_raw.csv"))

ratings_dL <- raw_ratings %>% group_by(participant,image) %>% gather(key = rating_type, value = response, arousal,valence,negativity,positivity,negativity)
```

## Ratings: Same Individual Sort

```
r1 <- ratings_dL %>%
  filter(grepl('M07',image)) %>%
  group_by(image, rating_type) %>%
  summarise(rating=ci(response)[1], 
            lowCI = ci(response)[2],
            hiCI = ci(response)[3], 
            sd = ci (response)[4]) %>%
  ggplot(aes(x=image,y=rating)) + 
  geom_bar(stat="identity") +
  geom_errorbar(aes(ymin=lowCI,ymax=hiCI),width=0,color="black")+
  scale_fill_brewer(palette="Set1")+
  facet_wrap(~rating_type)+
  theme(legend.position="none")+
  ggtitle("Same Individual Ratings") +
  theme(axis.text.x = element_text(angle = 90, hjust = 1,vjust=0.5))

r1
```

## Ratings: Different Individuals Sort

```
r2 <- ratings_dL %>%
  filter(!grepl('M07',image)) %>%
  group_by(image, rating_type) %>%
  summarise(rating=ci(response)[1], 
            lowCI = ci(response)[2],
            hiCI = ci(response)[3], 
            sd = ci (response)[4]) %>%
  ggplot(aes(x=image,y=rating)) + 
  geom_bar(stat="identity") +
  geom_errorbar(aes(ymin=lowCI,ymax=hiCI),width=0,color="black")+
  scale_fill_brewer(palette="Set1")+
  facet_wrap(~rating_type)+
  theme(legend.position="none")+
  ggtitle("Different Individual Ratings") +
  theme(axis.text.x = element_text(angle = 90, hjust = 1,vjust=0.5))

r2
```

## Range of responses

```
ratings_dL %>%
  ggplot(aes(x=response,fill=rating_type)) + 
  geom_bar(width = 0.5, color="black") +
  facet_wrap(~rating_type, strip.position="bottom") +
  theme(legend.position="none") +
  ggtitle("Range of rating responses")
```

## Correlation Tables of Dimension Ratings

```
cor1 <- ratings_pairs %>% filter(sort=="Sort1") %>% select(dist_valence,dist_arousal,dist_pos,dist_neg) %>% cor()
cor2 <- ratings_pairs %>% filter(sort=="Sort2") %>% select(dist_valence,dist_arousal,dist_pos,dist_neg) %>% cor()

cor1 <- as.data.frame(cor1) %>% rownames_to_column() %>% mutate(rowname=case_when(
  rowname=="dist_valence" ~ "Valence",
  rowname=="dist_arousal" ~ "Arousal",
  rowname=="dist_pos" ~ "Positivity",
  rowname=="dist_neg" ~ "Negativity")) %>% rename(" "=rowname)
cor1 %>% gt(cor1) %>% cols_align(align="center") %>% cols_label(dist_valence =md("Valence"), dist_arousal=md("Arousal"),dist_pos = md("Positivity"), dist_neg=md("Negativity")) %>%   
  tab_header(title = md("Same Individual Sort"))
```

| Same Individual Sort | | | | |
| --- | --- | --- | --- | --- |
|  | Valence | Arousal | Positivity | Negativity |
| --- | --- | --- | --- | --- |
| Valence | 1.0000000 | 0.38474924 | 0.9338119 | 0.85594920 |
| Arousal | 0.3847492 | 1.00000000 | 0.5589292 | 0.07386853 |
| Positivity | 0.9338119 | 0.55892920 | 1.0000000 | 0.64490296 |
| Negativity | 0.8559492 | 0.07386853 | 0.6449030 | 1.00000000 |

```
cor2 <- as.data.frame(cor2) %>% rownames_to_column() %>% mutate(rowname=case_when(
  rowname=="dist_valence" ~ "Valence",
  rowname=="dist_arousal" ~ "Arousal",
  rowname=="dist_pos" ~ "Positivity",
  rowname=="dist_neg" ~ "Negativity")) %>% rename(" "=rowname)
cor2 %>% gt(cor2) %>% cols_align(align="center") %>% cols_label(dist_valence =md("Valence"), dist_arousal=md("Arousal"),dist_pos = md("Positivity"), dist_neg=md("Negativity")) %>%   
  tab_header(title = md("Different Individual Sort"))
```

| Different Individual Sort | | | | |
| --- | --- | --- | --- | --- |
|  | Valence | Arousal | Positivity | Negativity |
| --- | --- | --- | --- | --- |
| Valence | 1.0000000 | 0.17383872 | 0.8986775 | 0.89707609 |
| Arousal | 0.1738387 | 1.00000000 | 0.2632563 | -0.03796251 |
| Positivity | 0.8986775 | 0.26325632 | 1.0000000 | 0.68076453 |
| Negativity | 0.8970761 | -0.03796251 | 0.6807645 | 1.00000000 |

# 8. Task Instruction Details

The study was divided into for phases (demo, practice, same individual sort and different individual sort).

Demo Sort: 1. Images on screen “Today we are going to play a game with pictures. We want to learn how these pictures go together” (pause to look at pictures) “We are going to start a game where we figure out where these different pictures should go.” 2. Sort Starts “In this game, we have all of these different boxes and we want to figure out which box each picture should go in. The rule of this game is that things that are of the same kind of thing go together and things that are different or not the same kind of thing go apart.”

Practice Sort (NO FEEDBACK): 1. Images on screen “Now we are going to try the same thing again but with new pictures.” (pause to look at pictures) 2. Sort Starts “Now we’re going to play again. Remember things that are of the same kind of thing go together and things that are different or not the same kind of thing go apart.”

Same Individual Sort (NO FEEDBACK): 1. Images on screen “Great job! Now we are going to play this game but with more pictures. This time, we want to sort the pictures of faces based on how people might feel inside. These are all faces of feelings. Can you see how some of these people might feel inside?” (pause to look at pictures) 2. Sort Starts “Now we’re going to play again. Remember people that feel the same kind of thing go together and people that feel a different kind of thing go apart.”

Different Individuals Sort (NO FEEDBACK): 1. Images on screen “Great job! Now we are going to play this game one last time. Remember, this time, we want to sort the pictures of faces based on how people might feel inside. These are all faces of feelings. Can you see how some of these people might feel inside?” (pause to look at pictures) 2. Sort Starts “Now we’re going to play one last time. Remember people that feel the same kind of thing go together and people that feel a different kind of thing go apart”

# Session Info

```
sessionInfo()
```

```
R version 4.1.1 (2021-08-10)
Platform: x86_64-apple-darwin17.0 (64-bit)
Running under: macOS Catalina 10.15.7

Matrix products: default
BLAS:   /Library/Frameworks/R.framework/Versions/4.1/Resources/lib/libRblas.0.dylib
LAPACK: /Library/Frameworks/R.framework/Versions/4.1/Resources/lib/libRlapack.dylib

locale:
[1] en_US.UTF-8/en_US.UTF-8/en_US.UTF-8/C/en_US.UTF-8/en_US.UTF-8

attached base packages:
[1] stats     graphics  grDevices utils     datasets  methods   base     

other attached packages:
 [1] gmodels_2.18.1     fossil_0.4.0       shapefiles_0.7     foreign_0.8-81    
 [5] maps_3.4.0         sp_1.4-5           ggrepel_0.9.1      gt_0.3.1          
 [9] broom_0.7.9        rmcorr_0.4.4       factoextra_1.0.7   cluster_2.1.2     
[13] ggcorrplot_0.1.3   corrplot_0.90      vegan_2.5-7        lattice_0.20-44   
[17] permute_0.9-5      dendextend_1.15.1  dendroextras_0.2.3 ggimage_0.2.9     
[21] ggdendro_0.1.22    harrietr_0.2.3     lmerTest_3.1-3     car_3.0-11        
[25] carData_3.0-4      lme4_1.1-27.1      Matrix_1.3-4       cowplot_1.1.1     
[29] here_1.0.1         forcats_0.5.1      stringr_1.4.0      dplyr_1.0.7       
[33] purrr_0.3.4        readr_2.0.1        tidyr_1.1.3        tibble_3.1.4      
[37] ggplot2_3.3.5      tidyverse_1.3.1    summarytools_1.0.0 knitr_1.34        

loaded via a namespace (and not attached):
  [1] readxl_1.3.1        backports_1.2.1     plyr_1.8.6         
  [4] lazyeval_0.2.2      splines_4.1.1       pryr_0.1.5         
  [7] digest_0.6.28       yulab.utils_0.0.4   htmltools_0.5.2    
 [10] viridis_0.6.1       magick_2.7.3        gdata_2.18.0       
 [13] fansi_0.5.0         magrittr_2.0.1      checkmate_2.0.0    
 [16] tzdb_0.1.2          openxlsx_4.2.4      modelr_0.1.8       
 [19] matrixStats_0.61.0  colorspace_2.0-2    rvest_1.0.1        
 [22] haven_2.4.3         xfun_0.26           tcltk_4.1.1        
 [25] crayon_1.4.1        jsonlite_1.7.2      ape_5.5            
 [28] glue_1.4.2          gtable_0.3.0        abind_1.4-5        
 [31] rapportools_1.0     scales_1.1.1        DBI_1.1.1          
 [34] Rcpp_1.0.7          viridisLite_0.4.0   tmvnsim_1.0-2      
 [37] gridGraphics_0.5-1  tidytree_0.3.6      httr_1.4.2         
 [40] RColorBrewer_1.1-2  ellipsis_0.3.2      pkgconfig_2.0.3    
 [43] farver_2.1.0        sass_0.4.0          dbplyr_2.1.1       
 [46] utf8_1.2.2          ggplotify_0.1.0     tidyselect_1.1.1   
 [49] labeling_0.4.2      rlang_0.4.11        munsell_0.5.0      
 [52] cellranger_1.1.0    tools_4.1.1         cli_3.0.1          
 [55] generics_0.1.0      evaluate_0.14       fastmap_1.1.0      
 [58] yaml_2.2.1          ggtree_3.2.0        fs_1.5.0           
 [61] zip_2.2.0           pander_0.6.4        nlme_3.1-152       
 [64] aplot_0.1.1         xml2_1.3.2          compiler_4.1.1     
 [67] rstudioapi_0.13     curl_4.3.2          reprex_2.0.1       
 [70] treeio_1.18.1       bslib_0.3.0         stringi_1.7.4      
 [73] highr_0.9           commonmark_1.7      psych_2.1.9        
 [76] nloptr_1.2.2.2      vctrs_0.3.8         pillar_1.6.2       
 [79] lifecycle_1.0.0     jquerylib_0.1.4     data.table_1.14.0  
 [82] patchwork_1.1.1     R6_2.5.1            gridExtra_2.3      
 [85] rio_0.5.27          codetools_0.2-18    gtools_3.9.2       
 [88] boot_1.3-28         MASS_7.3-54         assertthat_0.2.1   
 [91] rprojroot_2.0.2     withr_2.4.2         mnormt_2.0.2       
 [94] mgcv_1.8-36         parallel_4.1.1      hms_1.1.0          
 [97] grid_4.1.1          ggfun_0.0.4         minqa_1.2.4        
[100] rmarkdown_2.11      numDeriv_2016.8-1.1 lubridate_1.7.10   
[103] base64enc_0.1-3
```
